# *Cordyceps cicadae* and *Cordyceps gunnii* have closer species correlation with *Cordyceps sinensis*: from the perspective of metabonomic and MaxEnt models

**DOI:** 10.1038/s41598-022-24309-z

**Published:** 2022-11-28

**Authors:** Min Zhang, Xiao Sun, Yujing Miao, Minhui Li, Linfang Huang

**Affiliations:** 1 A Key Laboratory of Chinese Medicine Resources Conservation, State Administration of Traditional Chinese Medicine of the People’s Republic of China, Institute of Medicinal Plant Development, Chinese Academy of Medical Sciences & Peking Union Medical College, Beijing, 100193 China; 2 College of Pharmacy, Baotou Medical College, Baotou, 014040 China; 3 Inner Mongolia Hospital of Traditional Chinese Medicine, Hohhot, 010020 China

**Keywords:** Computational biology and bioinformatics, Plant sciences

## Abstract

*Cordyceps sinensis* is a second-class nationally-protected medicinal fungus and functional food. *Cordyceps sinensis* resources are endangered, and finding new medicinal materials is a fast and economical way to meet the current demonstrated demand, which can effectively solve the shortage of *C. sinensis* resources. In this study, the metabolite characteristics of *Cordyceps* were comprehensively revealed by LC-QTOF-MS technology. The maxent model can be used to predict the habitat suitability distribution of *Cordyceps* and screen out the main climatic factors affecting its distribution. The correlation model between climate factors and chemical components was established by Pearson correlation analysis. Finally, based on the analysis of climate factors and metabolites, we will analyze the high correlation species with *C. sinensis*, and develop them as possible alternative species of *C. sinensis* in the future. The results showed that the suitable area of *Cordyceps cicadae* demonstrated a downward trend, while that of *C. sinensis, Cordyceps militaris and Cordyceps gunnii* demonstrated an upwards trend. The suitable areas all shifted to the northwest. The temperature seasonality and max temperature of the warmest month are the maximum climatic factors affecting nucleosides. Compared with *C. sinensis*, the metabolic spectrum similarities of *C. cicadae*, *C. militaris*, and *C. gunnii* were 94.42%, 80.82%, and 91.00%, respectively. *Cordyceps sinensis, C. cicadae, and C. gunnii* were correlated well for compounds and climate factors. This study will explore whether *C. cicadae, C. militaris* and *C. gunnii* can be used as substitutes for *C. sinensis*. Our results may provide a reference for resource conservation and sustainable utilization of endangered *C. sinensis*.

## Introduction

*Cordyceps sinensis* is a rare medicinal fungus in China and is listed as a second-class national key protected wild plant^1,2^. *Cordyceps sinensis* has the therapeutic effect of regulating the human immune system and its antitumor and antioxidation activities, and it can improve cachexia and prolong life. It is worth mentioning that in recent years, as *C. sinensis* has become a favoured functional food, its market demand has increased yearly. Due to its harsh habitat and excessive digging, wild *C. sinensis* resources are in short supply, and the IUCN red list status is vulnerable^3,4^. Therefore, finding new resources for medicinal material is a fast and economical way to meet the current demonstrated needs, thereby effectively compensating for the scarce resources of this rare Chinese traditional medicine. More than 350 species of *Cordyceps* have been reported worldwide. Of these species, only a few, such as *Cordyceps sinensis* (Berk.) Sacc., *Cordyceps sobolifera* (Hill.) Berk., C*ordyceps ophioglossides* (Ehr.) Link. and *Cordyceps militaris* (Vuill.) Fr., are used for tonics and therapeutic drugs^5^. The *National Compilation of Chinese Herbal Medicine* states: "*Cordyceps militaris* fruit body and insect body can also be used as *C. sinensis* medicine"^6^. In 1983, the asexual form of *Cordyceps gunnii* was first reported in China as a new species of *Paecilomyces*, and the mycelia of *Paecilomyces gunnii* had UV absorption spectra similar to those of *C. sinensis*^7^. *Cordyceps cicadae* is a kind of fungal TCM that is included in the *Traditional Chinese Medicines in Zhejiang Province of Processing Chinese Crud Drugs*^8^*.* There is sufficient evidence that the nucleosides and some biological activities of *C. cicadae* are similar to those of *C. sinensis*^9–12^. The medicinal values and health care effects of *C. militaris*, *C. cicadae* and *C. gunnii* indicate that they are expected to become substitutes for *C. sinensis*^13^. In addition, the cultivation and development conditions of *C. militaris*, *C. cicadae* and *C. gunnii* are not as harsh as those of *C. sinensis*, so they can be used as ideal new drug sources for the research and development of *C. sinensis*.

At present, there are many studies on the biologically active components of *C. sinensis*. Some metabolites (including cordycepin, cordycepic acid, several other nucleosides, cyclic peptides, sterols and polysaccharides) isolated from *Cordyceps* and the pharmacological properties of some species have been verified in vitro and in vivo^14–17^, demonstrating the phytochemical diversity of *Cordyceps* and the strong biological activity of these components, which can be used as possible clues for drug discovery^18,19^. However, in addition to studies in the chemical and pharmacological fields of TCM resources, the habitat of *Cordyceps* species has undergone profound changes under global warming and anthropogenic pressure, leading to a drastic decline in its wild population^20,21^. We found that there are limited studies to predict the future geographical distribution of *Cordyceps* species and their correlation with chemical composition. In this study, the metabolic profiles of *C. sinensis*, *C. militaris*, *C. cicadae* and *C. gunnii* were comprehensively and directly analysed by extensive targeted metabolomics. The ecological environment, which determines the main factors affecting the quality and source of Chinese medicinal materials, has always been the focus of research. The synthesis and accumulation of the effective components of TCM are closely related to the ecological environment^22^. As one of the ecological environmental factors, climatic conditions have diverse and complex effects on the quality of TCM^22–24^. MaxEnt model is a prediction scheme of species geographical distribution which integrates niche theory, occurrence data model and maximum entropy principle. The data selected by maxent include: (1) location data of specific species (latitude and longitude, the presence or absence of species); (2) the necessary environmental conditions for the survival of the species at the same georeferential point. The environmental layers commonly used for modeling are temperature and rainfall. After the simulation is generated, the resulting probability function is applied to the interesting geographic area to form the output file containing the occurrence probability of the species. MaxEnt is a statistical method based on known species distribution information and environmental data that is used to predict unknown distributions^25^. Compared with genetic algorithm for ruleset production (GARP), random forest (RF) and other models, MaxEnt has a relatively simple modeling process, a high tolerance for sample quantity and quality, and the prediction results are excellent^25^. It is one of the most popular models in recent years in the field of predicting species-suitable areas^26–28^. Of course, the MaxEnt model has some limitations, such as besides climate variables, other factors including soil, topography, biotic factors and human activity also affect the content of active compounds in herbs and are key to the formation of metabolites in medicinal plants. Further research on incorporating these factors into models, would further enhance predictions about likely changes in the distribution of *Cordyceps* in China as a result of changing climate conditions. Based on the nonrandom relationship between climate factors of species, spatial distribution data and the study area, under certain limiting conditions, the maximum probability distribution was found to be the optimal distribution, the suitable area of species was predicted, and the spatial distribution model of its geographical scale was constructed^28–31^. Previous studies have combined species geographical distribution prediction and chemical composition analysis^32–34^, for example, Wan et al. established a new method to evaluate the impact of environmental factors on the quality of *Codonopsis pilosula* based on ultra-high performance chromatographic (UPLC) fingerprint technology and MaxEnt model^32^. Li et al. used maxent model and chemical analysis to model the distribution of potential species of *Coptis* herbs as a function of environmental variables and altitude^33^. Sun et al. studied the quality ecotypes of *Panax quinquefolium* based on genetic, chemical and ecological characteristics^34^. Based on the above examples, we believe that learning based on MaxEnt model and metabolome technology is necessary and effective, and these studies provide reasonable basis for habitat suitability assessment and resource conservation of endangered medicinal plants. Inspired by previous researches and the existing scientific research foundation of our team, this study makes the following research hypotheses: (1) The metabolites of *Cordyceps* may be affected by climatic conditions, and the correlation between metabolites and climatic factors should be further explored; (2) Based on climate factors and metabolites, we sought alternative species with high correlation with *C. sinensis* in *Cordyceps* genus. In order to expand the medicinal resources and provide a reasonable protective strategy for *C. sinensis*.

In conclusion, this study used widely targeted metabolomics and the MaxEnt model as technical means and nucleoside metabolites and climate factors as evaluation indices, combined with multivariate statistical analysis, to evaluate the species correlation between *C. sinensis* and *C. militaris*, *C. cicadae*, and *C. gunnii*.

## Results

### Principal component analysis (PCA)

**Figure 1:**
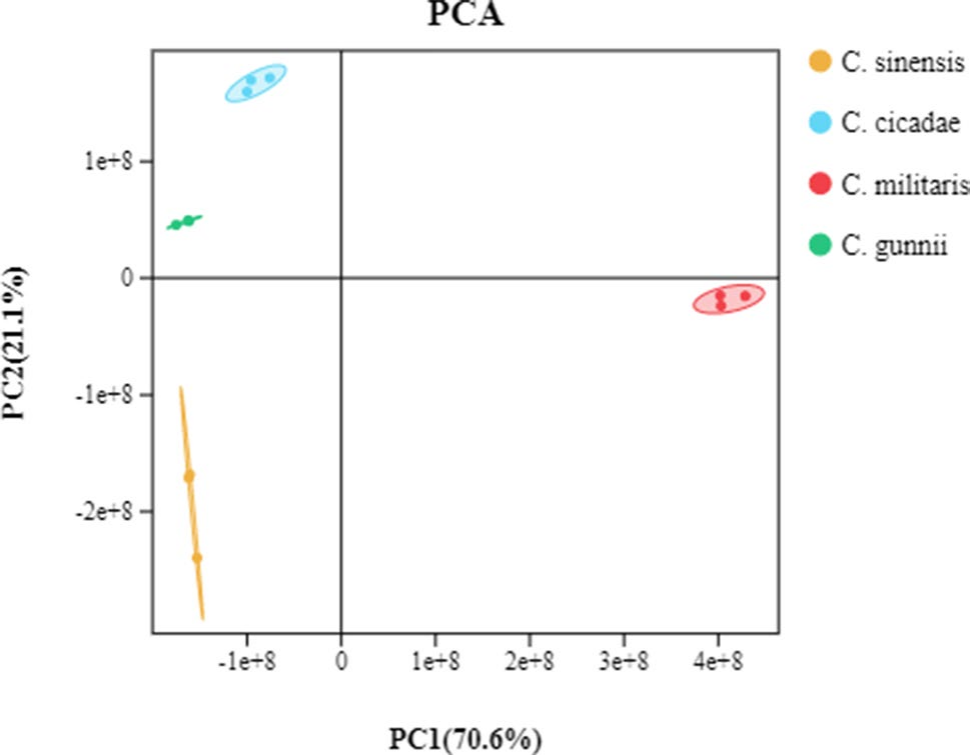
PCA results of *C. sinensis*, *C. cicadae*, *C. militaris* and *C. gunnii* samples and quality control.

**Figure 2:**
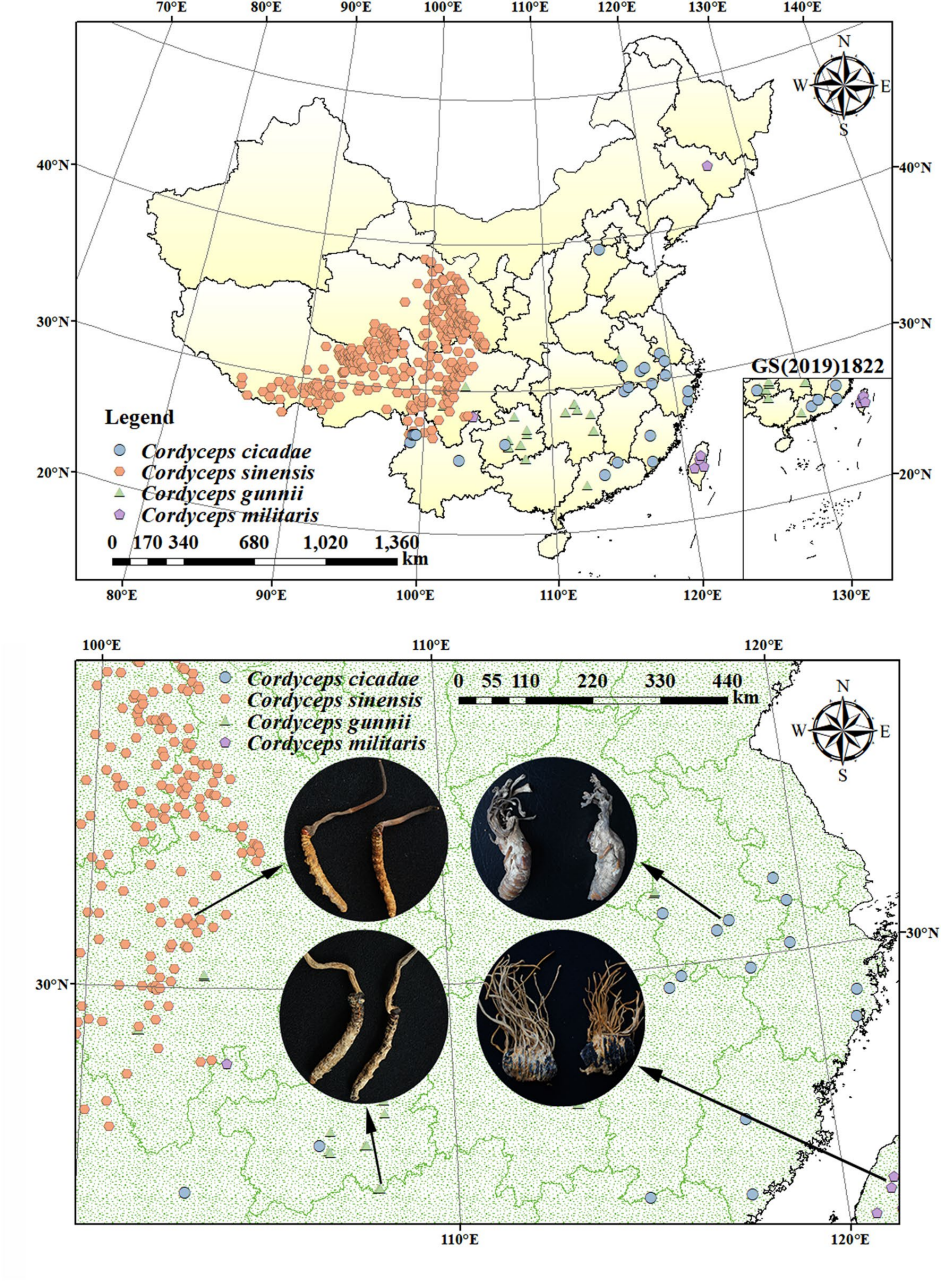
Species distribution point and sample photographs of *C. sinensis*, *C. cicadae*, *C. militaris* and *C. gunnii*. The figure was accomplished by ArcGIS (version 10.7, https://www.esri.com/zh-cn/arcgis/) and MaxEnt software (version 3.4.1, https://biodiversityinformatics.amnh.org/open_source/maxent/).

The results of PCA showed that (Fig. 1) the contribution rate of PC1 was 65% and that of PC2 was 20.1%. The cumulative contribution rate of PC1 and PC2 reached 85.1%. According to the dispersion analysis of sample repeatability and difference, the stability of the instrument and the validity of the data were determined. If the sample dispersion is small, the instrument is stable, and the test result is reliable. As shown in Fig. 2, the four sample groups are obviously separated, and the inserted mixed QC samples overlap well, indicating that the instrument has good stability. Moreover, the small dispersion of biological repeat samples within the sample group indicates that the biological repeat samples have good repeatability and representativeness, indicating feasibility for the accuracy of subsequent metabolite analysis results.

### Orthogonal partial least squares discriminant analysis (OPLS-DA)

**Figure 3:**
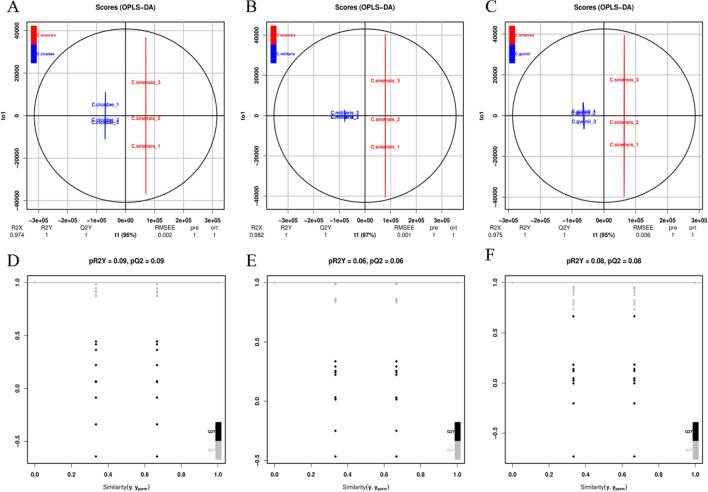
OPLS-DA model score diagram and model verification diagram of *C. sinensis*, *C. cicadae*, *C. militaris* and *C. gunnii* samples. (**A**) Score diagram of OPLS-DA model between *C. sinensis* and *C. cicadae*. (**B**) Score diagram of OPLS-DA model between *C. sinensis* and *C. militaris*. (**C**) Score diagram of OPLS-DA model between *C. sinensis* and *C. gunnii*. (**D**) OPLS-DA model verification diagram between *C. sinensis* and *C. cicadae*. (**E**) OPLS-DA model verification diagram between *C. sinensis* and *C. militaris*. (**F**) OPLS-DA model verification diagram between *C. sinensis* and *C. gunnii*.

In contrast to principal component analysis (PCA) and orthogonal partial least-squares discriminant analysis (OPLS-DA), which is another kind of supervised learning method^35^, this method can successfully separate samples and is more conducive to finding different metabolites. The OPLS-DA model score figures (Fig. 3A–C) of *C. sinensis* and *C. cicadae*, *C. militaris,* and *C. gunnii* found that groups of samples can be gathered into a class. The difference between the two groups was obvious, indicating that the experiment had good repeatability and that the results were similar to those of PCA.

In addition, R^2^X = 0.974, R^2^Y = 1, and Q^2^Y = 1 for *C. sinensis* and *C. cicadae*; R^2^X = 0.982, R^2^Y = 1, and Q^2^Y = 1 for *C. sinensis* and *C. militaris*; R^2^X = 0.975 and R^2^Y = 1 for *C. sinensis* and *C. gunnii*; and Q^2^Y = 1. These three indicators are close to (or equal to) 1, indicating that the more stable and reliable the OPLS-DA model is, the better it can predict the different metabolites of *C. sinensis* and *C. cicadae*, *C. militaris*, and *C. gunnii*. To prevent overfitting of the OPLS-DA model, we adopted the substitution test method to verify the OPLS-DA model. From Fig. 3D–F, the OPLS-DA model established by the metabolite data in this experiment was not fitted. In our study, variable important in projection (VIP) was used to measure the influence strength and explanatory ability of metabolite accumulation differences on the classification and discrimination of each group of samples. VIP ≥ 1 was a common screening standard for differential metabolites, and the larger the VIP value was, the greater the contribution to the classification of the OPLS-DA model, and the greater the difference in the content of this compound between the two groups. A total of 852 different metabolites were preliminarily detected in the three groups, among which 294 different metabolites were detected between *C. sinensis* and *C. cicadae*, 188 of which were significantly upregulated and 106 of which were significantly downregulated. A total of 284 different metabolites were detected between *C. sinensis* and *C. militaris*. A total of 173 metabolites were significantly upregulated, and 111 metabolites were significantly downregulated. A total of 274 different metabolites were detected between *C. sinensis* and *C. gunnii*, 142 of which were significantly upregulated and 132 of which were significantly downregulated.

### Comparative analysis of metabolites

**Figure 4:**
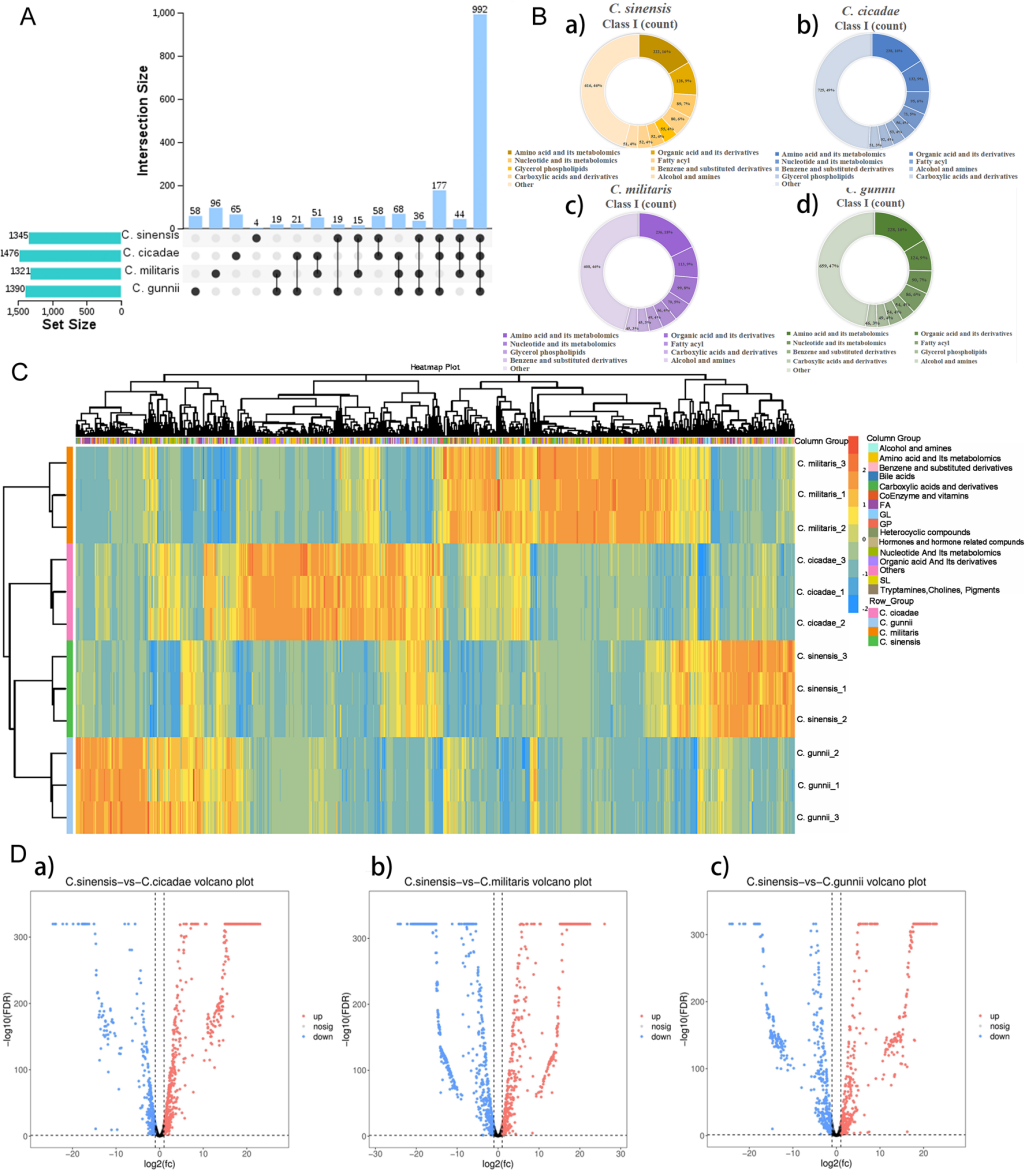
Analysis of total metabolites in *C. sinensis, C. cicadae, C. militaris and C. gunnii*. (**A**) UpSet venn diagram for the total difference of metabolites between *C. sinensis, C. cicadae, C. militaris and C. gunnii*. (**B**) Pie chart of grouping proportion of total metabolites in the samples of *C. sinensis* (a)*, C. cicadae* (b), *C. militaris* (c)*, C. gunnii* (d). (**C**) Cluster heat map of total metabolites in the samples of *C. sinensis, C. cicadae, C. militaris and C. gunnii*. (**D**) Volcanic map of total different metabolites between *C. sinensis* and *C. cicadae* (a), *C. militaris* (b), *C. gunnii* (c).

Sixteen types of metabolites were detected in *Cordyceps* fungi based on the LC-QTOF-MS method, among which 1345 metabolites were identified in *C. sinensis* (Fig. 4A,B). The types of compounds in the top three included 222 amino acids and their metabolites (16%), 128 organic acids and their derivatives (9%) and 89 nucleotides and their metabolites (7%). A total of 1476 metabolites were identified from *C. cicadae*. The types of compounds in the top three included 238 kinds of amino acids and their metabolites (16%), 132 kinds of organic acids and their derivatives (9%) and 95 kinds of nucleotides and their metabolites (6%). A total of 1321 metabolites were identified from *C. militaris*. The types of compounds in the top three included 236 kinds of amino acids and their metabolites (18%), 113 kinds of organic acids and their derivatives (9%) and 99 kinds of nucleotides and their metabolites (8%). A total of 1390 metabolites were identified from *C. gunnii*. The types of compounds in the top three included 228 kinds of amino acids and their metabolites (16%), 124 kinds of organic acids and their derivatives (9%) and 90 kinds of nucleotides and their metabolites (7%). Compared with *C. sinensis* and *C. cicadae*, *C. militaris*, and *C. gunnii*, the similarity of the metabolic spectrum was 94.42%, 80.82%, and 91.00%, respectively.

The *p value* or fold change of univariate analysis was used to further screen out the differential metabolites in combination with univariate statistical analysis. Generally, fold change ≥ 2 and fold change ≤ 0.5 or *p value* < 0.05 were selected, and the above two were used as screening criteria (Fig. 4D). A total of 204 metabolites were detected, among which 68 different metabolites were detected between *C. sinensis* and *C. cicadae*; 47 metabolites were significantly upregulated, and 21 metabolites were significantly downregulated. A total of 66 different metabolites were detected between *C. sinensis* and *C. militaris*, with 43 metabolites significantly upregulated and 23 metabolites significantly downregulated. A total of 70 different metabolites were detected between the two groups of *C. sinensis* and *C. gunnii*, of which 50 metabolites were significantly upregulated and 20 were significantly downregulated.

To more clearly see the differences in compounds in *C. sinensis, C. cicadae, C. militaris and C. gunnii*, the range method was used to carry out the normal processing of the metabolite content data of the four species of *Cordyceps*, for which R software (http://www.r-project.org/) was used. Hierarchical cluster analysis (HCA) was conducted for metabolite accumulation patterns among different samples. As shown in Fig. 4C, each rectangle in the heatmap represents a metabolite whose content is coloured according to a standardized scale of − 2.0 (low) to 2.0 (high), with red representing higher than average compound relative content and blue representing lower than average compound relative content. The tree shows the presence of different subclusters, including different numbers of metabolites with varying degrees of similarity, as well as biochemically related compounds commonly found clustered together; the compounds are represented horizontally, and the four species of cordyceps, vertically.

**Figure 5:**
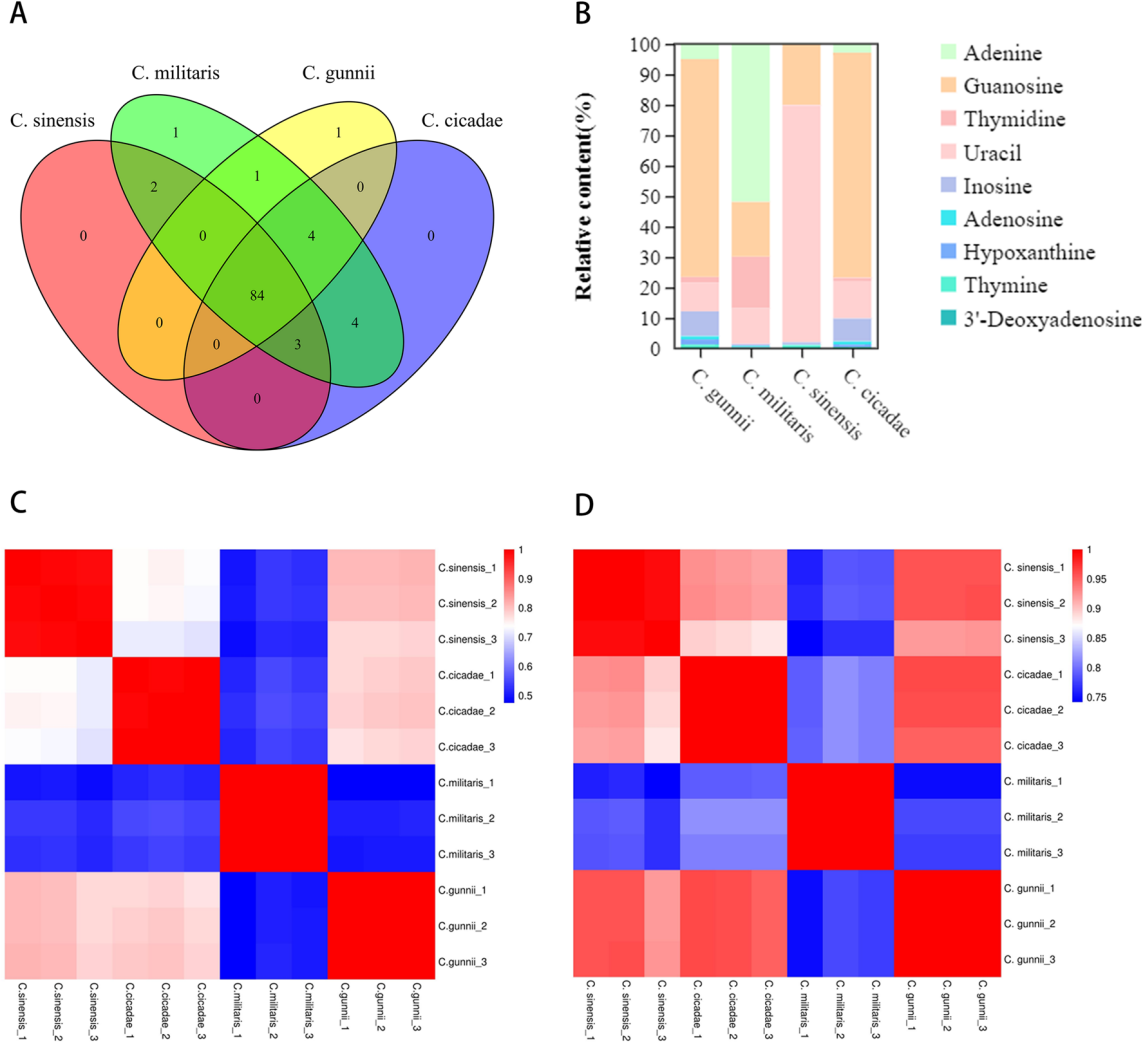
Analysis of nucleoside metabolites of Cordyceps. (**A**) venn diagram of nucleoside metabolites of *C. sinensis, C. cicadae, C. militaris* and *C. gunnii*. (**B**) Histogram of the relative contents of major components in nucleoside metabolites of *C. sinensis, C. cicadae, C. militaris* and *C. gunnii*. (**C**) Pearson correlation heat map of all metabolites of *C. sinensis, C. cicadae, C. militaris* and *C. gunnii.* (**D**) Pearson correlation heat map of nucleoside metabolites of *C. sinensis, C. cicadae, C. militaris* and *C. gunnii.*

Pearson correlation analysis was performed for all metabolites detected by metabolomics techniques (Fig. 5C). The results of the heatmap showed that a *p value* < 0.05 for *C. sinensis, C. cicadae, C. militaris and C. gunnii* within and between groups, which represented the significant correlation between samples within and between groups. The correlation coefficient (r) was used to further evaluate the degree of correlation. The closer it was to 1, the stronger the correlation. The correlation coefficient between *C. sinensis and C. gunnii* ranged from 0.8 to 1.0, showing a strong positive correlation. The correlation coefficients between *C. sinensis and C. cicadae*, *C. gunnii and C. cicadae* were between 0.6 and 0.8, also showing a strong positive correlation. The correlation coefficients of *C. sinensis and C. militaris*, *C. militaris* and *C. cicadae*, *C. militaris* and *C. gunnii* were in the range of 0.4–0.6, showing a moderate positive correlation.

**Figure 6:**
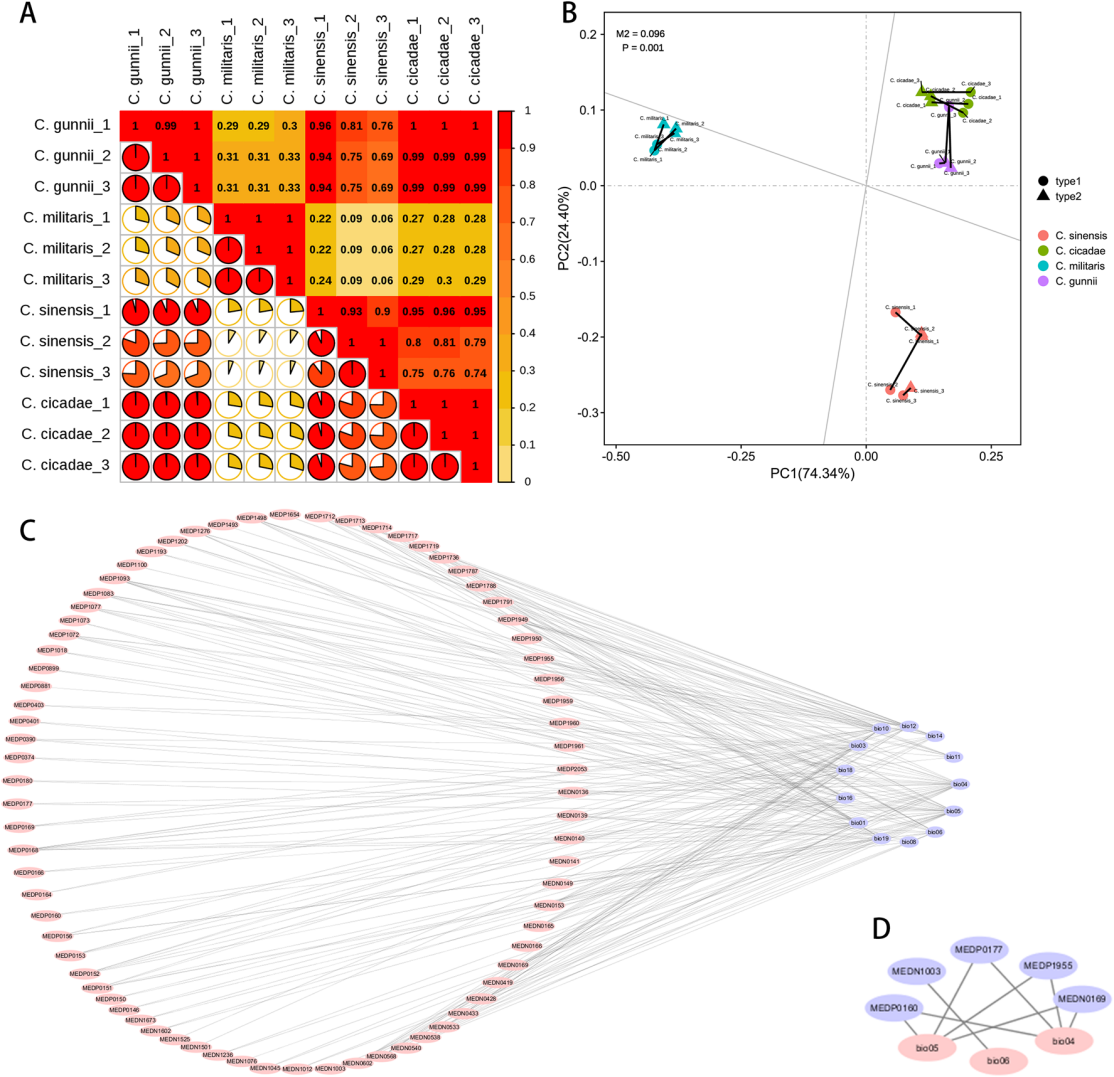
(**A**) Analyze the interspecific correlation heat map of *C. sinensis*, *C. cicadae*, *C. militaris* and *C. gunnii* according to ecological climate factors. (**B**) The results of the Platts analysis of ecoclimate factors and nucleoside metabolites. (type1: ecoclimate factors, type2: nucleoside metabolites.) (**C**) Network diagram of correlations between eco-climate factors and nucleoside metabolites. (**D**) Correlation network diagram of eco-climate factors and major components in nucleoside metabolites.

Many bioactivity studies reported for the species belonging to the *Cordyceps* genus have been associated with the presence of nucleosides. The majority of the nucleosides reported from *Cordyceps* species were isolated from *C. sinensis*, *C. militaris* and *C. cicadae*. These nucleosides display potent anticancer, antiviral, neuroprotective, anti-inflammatory, antitumor and antioxidant activities^36^. By comparing the number of nucleoside metabolites among *C. sinensis, C. cicadae, C. militaris and C. gunnii* (Fig. 5A), it can be seen that there are 84 species of nucleoside metabolites in *C. sinensis, C. cicadae, C. militaris and C. gunnii*, and there is 1 specific nucleoside compound in both *C. militaris* and *C. gunnii*. Pearson correlation analysis results of all nucleosides (Fig. 5D) were ranked from strong to weak, and the correlation coefficients between various groups, *C. gunnii and C. cicadae*, *C. sinensis and C. gunnii*, *C. sinensis and C. cicadae*, and *C. militaris* and *C. cicadae,* were in the range of 0.8–1.0, showing a strong positive correlation. The correlation coefficients between *C. sinensis and C. militaris* and between *C. militaris* and *C. gunnii* were between 0.6 and 0.8, showing a strong positive correlation. According to the pearson correlation heatmap (Supplementary Fig. S2) analysis of the 9 principal components of nucleoside metabolites, there was a *p value* < 0.05 for *C. sinensis, C. cicadae, C. militaris*, *C. gunnii* groups, *C. gunnii and C. cicadae*, and the correlation coefficient (r) was > 0.99, indicating that the correlation among all groups was significant, strong and positive. The correlation between the other sample groups was in the order of strong to weak: *C. sinensis and C. cicadae*, *C. sinensis and C. gunnii*, *C. militaris* and *C. gunnii, C. militaris* and *C. cicadae*, *C. sinensis and C. militaris*. As shown in Fig. 6C, the relative contents of the 9 main components of nucleoside metabolites of *C. sinensis, C. cicadae, C. militaris and C. gunnii* decreased in the order of adenosine > guanosine > inosine > hypoxanthine > thymine > 3′-deoxyadenosine > uracil > thymidine > adenine from large to small. This result is consistent with the conclusion that adenosine is the effective, determining component in the content of *C. sinensis* in the “Chinese pharmacopoeia”. As shown in Table 1, the nucleoside compounds detected in different species of the same cordyceps were different, and their relative content was also different. The relative contents of nucleoside compounds in *C. sinensis, C. cicadae, C. militaris and C. gunnii* were 2.22E+09 ± 1.60E+08, 2.12E+09 ± 1.08E+08, 2.80E+09 ± 8.99E+07 and 1.82E+09 ± 6.95E+07, respectively. However, the relative content of adenosine (Fig. 5B) was in the order of *C. sinensis* > *C. gunnii* > *C. cicadae* > *C. militaris.* The results showed that both *C. gunnii* and *C. cicadae* demonstrated a strong correlation with *C. sinensis* in total metabolites and nucleoside compounds. *Cordyceps gunnii* and *C. cicadae* are expected to be medicinal substitutes for *C. sinensis* to develop new medicinal sources and alleviate the problem of *C. sinensis* resource depletion.

**Table 1:** Relative contents of nucleosides in *C. sinensis*, *C. cicadae*, *C. militaris* and *C. gunnii* were determined by LC-QTOF-MS/MS.

Index	Compounds	Ionization model	Formula	*C. sinensis*		*C. cicadae*		*C. militaris*		*C. gunnii*	
				Mean	Standard deviation	Mean	Standard deviation	Mean	Standard deviation	Mean	Standard deviation
MEDN0136	1,7-Dimethylxanthine	[M−H]^−^	C_7_H_8_N_4_O_2_	9	0	9	0	1.23E+05	1.58E+04	9	0
MEDN0139	1-Methylxanthine	[M−H]^−^	C_6_H_6_N_4_O_2_	1.84E+05	1.14E+04	1.55E+06	6.72E+04	1.03E+05	1.40E+04	1.40E+05	1.25E+04
MEDN0140	Xanthine	[M−H]^−^	C_5_H_4_N_4_O_2_	6.40E+04	3.37E+04	5.78E+05	7.18E+04	3.01E+05	1.02E+05	1.09E+06	2.68E+05
MEDN0141	2′-Deoxyuridine	[M−H]^−^	C_9_H_12_N_2_O_5_	5.05E+04	5.83E+03	2.58E+05	1.19E+04	6.87E+05	4.54E+04	2.74E+05	2.50E+04
MEDN0149	7-Methylxanthine	[M−H]^−^	C_6_H_6_N_4_O_2_	3.80E+04	6.30E+03	3.04E+05	1.64E+04	2.50E+04	3.87E+03	4.04E+04	8.47E+03
MEDN0151	Adenine	[M−H]^−^	C_5_H_5_N_5_	9.00E+00	0.00E+00	8.13E+04	4.14E+04	2.74E+05	5.95E+04	2.66E+05	2.97E+04
MEDN0153	Adenosine 5′-monophosphate	[M−H]^−^	C_10_H_14_N_5_O_7_P	5.83E+06	1.25E+06	1.31E+06	2.04E+05	3.43E+06	6.09E+05	2.63E+05	9.09E+04
MEDN0160	Guanosine	[M−H]^−^	C_10_H_13_N_5_O_5_	5.44E+07	3.43E+06	9.60E+07	9.70E+06	8.82E+07	4.11E+06	4.51E+07	1.40E+06
MEDN0165	Inosine 5′-monophosphate	[M−H]^−^	C_10_H_13_N_4_O_8_P	4.42E+05	9.55E+04	1.04E+05	1.95E+04	2.42E+05	2.03E+04	2.01E+04	5.48E+03
MEDN0166	Nicotinic acid adenine Dinucleotide	[M−H]^−^	C_21_H_27_N_7_O_14_P_2_	3.64E+04	2.09E+04	1.55E+04	5.90E+03	1.10E+05	2.37E+04	1.88E+04	9.63E+03
MEDN0168	Thymidine	[M−H]-	C_10_H_14_N_2_O_5_	4.20E+04	7.04E+03	9.02E+04	1.63E+04	1.12E+05	4.73E+03	1.56E+05	8.06E+03
MEDN0169	Uracil	[M−H]^−^	C_4_H_4_N_2_O_2_	7.16E+04	8.61E+03	4.64E+04	8.74E+03	3.03E+05	4.53E+04	3.94E+04	1.84E+04
MEDN0171	Uridine	[M−H]^−^	C_9_H_12_N_2_O_6_	6.09E+04	1.72E+04	1.04E+05	2.34E+04	1.04E+05	2.20E+04	1.17E+05	1.49E+04
MEDN0306	Creatine phosphate	[M+H]^+^	C_4_H_10_N_3_O_5_P	1.06E+05	2.47E+04	7.88E+04	4.07E+03	6.42E+04	6.39E+03	2.02E+05	3.49E+04
MEDN0419	Uridine 5-monophosphate	[M−H]^−^	C_9_H_13_N_2_O_9_P	5.15E+06	9.07E+05	1.38E+06	2.25E+05	6.12E+05	2.34E+05	9	0
MEDN0428	N6-succinyl adenosine	[M−H]^−^	C_14_H_17_N_5_O_8_	2.46E+05	4.87E+03	3.03E+05	3.78E+04	1.21E+05	8.27E+03	2.33E+05	1.08E+04
MEDN0431	2-(Dimethylamino)guanosine	[M−H]^−^	C_12_H_17_N_5_O_5_	1.34E+04	1.01E+03	2.58E+06	8.66E+04	2.10E+06	5.73E+04	1.34E+05	4.11E+03
MEDN0433	8-Hydroxy-2-deoxyguanosine	[M−H]^−^	C_10_H_13_N_5_O_5_	1.22E+05	2.42E+04	6.40E+04	2.71E+03	4.27E+04	4.98E+03	5.95E+04	1.14E+04
MEDN0452	2′-Deoxycytidine-5′-monophosphate	[M−H]^−^	C_9_H_14_N_3_O_7_P	7.09E+04	2.02E+04	1.01E+05	1.86E+04	1.01E+05	1.38E+03	2.68E+05	9.46E+03
MEDN0533	Xanthosine	[M−H]^−^	C_10_H_12_N_4_O_6_	3.36E+05	3.50E+04	4.31E+06	6.45E+05	3.73E+06	3.09E+05	7.25E+06	2.12E+05
MEDN0537	ADP-ribose	[M−H]^−^	C_15_H_23_N_5_O_14_P_2_	5.70E+04	8.33E+03	1.18E+04	3.12E+03	4.59E+04	2.79E+04	2.01E+04	1.46E+04
MEDN0538	UDP-glucose	[M−H]^−^	C_15_H_24_N_2_O_17_P_2_	1.10E+05	2.57E+04	4.66E+04	8.69E+03	5.80E+06	9.84E+05	9	0
MEDN0540	1-Methylguanine	[M−H]^−^	C_6_H_7_N_5_O	1.20E+04	2.64E+03	1.88E+05	1.72E+04	3.01E+04	2.71E+03	2.33E+04	3.60E+03
MEDN0568	2-Methylguanosine	[M−H]^−^	C_11_H_15_N_5_O_5_	3.76E+04	4.74E+03	1.05E+06	5.08E+04	8.88E+05	9.04E+04	1.08E+06	4.09E+04
MEDN0602	Deoxyguanosine 5′-monophosphate (dGMP)	[M−H]^−^	C_10_H_14_N_5_O_7_P	5.83E+06	1.25E+06	1.31E+06	2.04E+05	3.43E+06	6.09E+05	2.63E+05	9.09E+04
MEDN1003	Inosine	[M−H]^−^	C_10_H_12_N_4_O_5_	2.66E+07	2.00E+06	8.31E+07	7.02E+06	3.14E+07	1.25E+06	2.82E+07	4.75E+05
MEDN1012	7-Methylguanine	[M−H]^−^	C_6_H_7_N_5_O	1.20E+04	2.64E+03	1.88E+05	1.72E+04	3.01E+04	2.71E+03	2.33E+04	3.60E+03
MEDN1013	2′-Deoxyadenosine-5′-monophosphate	[M−H]^−^	C_10_H_14_N_5_O_6_P	5.74E+04	8.20E+03	1.08E+05	1.04E+04	1.48E+05	2.83E+04	2.01E+05	1.83E+05
MEDN1045	2-Amino-4,6-pteridinediol	[M−H]^−^	C_6_H_5_N_5_O_2_	9	0	5.25E+04	1.80E+03	3.40E+05	6.75E+04	9	0
MEDN1076	N6-isopentene adenine	[M−H]^−^	C_10_H_13_N_5_	9	0	4.44E+04	3.57E+03	7.68E+04	3.63E+03	9	0
MEDN1236	Cyclic ADP ribose	[M−H]^−^	C_15_H_21_N_5_O_13_P_2_	4.62E+04	2.33E+04	1.61E+04	2.36E+03	8.45E+04	1.53E+04	2.38E+04	1.14E+04
MEDN1501	2′-O-methyluridine	[M−H]^−^	C_10_H_14_N_2_O_6_	8.86E+03	2.91E+03	5.32E+04	5.42E+03	6.88E+04	8.72E+03	4.09E+04	2.40E+03
MEDN1513	5-Hydroxy-2′-deoxyuridine	[M−H]^−^	C_9_H_12_N_2_O_6_	6.09E+04	1.72E+04	1.04E+05	2.34E+04	1.04E+05	2.20E+04	1.17E+05	1.49E+04
MEDN1515	5-Hydroxymethyl-2′-deoxycytidine	[M−H]^−^	C_10_H_15_N_3_O_5_	9	0	9	0	9	0	3.58E+05	6.47E+04
MEDN1525	Coproporphyrin III	[M−H]^−^	C_36_H_38_N_4_O_8_	9	0	2.06E+04	5.91E+03	3.12E+04	2.55E+03	1.52E+04	3.22E+03
MEDN1529	Cytidine 5′-monophosphate-N-acetylneuraminic acid (CMP-NANA)	[M−H]^−^	C_20_H_31_N_4_O_16_P	1.22E+04	7.59E+03	5.32E+03	2.62E+03	4.75E+03	1.96E+03	1.45E+04	8.47E+03
MEDN1586	Thymidine-5′-phosphate (dTMP)	[M−H]^−^	C_10_H_15_N_2_O_8_P	1.65E+05	3.83E+04	6.07E+05	4.13E+04	3.84E+05	6.48E+04	5.29E+05	4.86E+05
MEDN1602	3-Deoxyguanosine	[M−H]^−^	C_10_H_13_N_5_O_4_	1.88E+06	4.43E+04	5.00E+06	4.09E+05	1.26E+07	4.84E+05	4.24E+05	4.82E+04
MEDN1673	6-Benzylaminopurine	[M−H]^−^	C_12_H_11_N_5_	9	0	9	0	1.32E+04	2.39E+03	1.40E+03	2.40E+03
MEDN1675	2-Deoxyribose-5′-phosphate	[M−H]^−^	C_5_H_11_O_7_P	7.55E+04	1.37E+04	5.43E+04	8.83E+03	7.97E+04	2.23E+04	5.13E+04	1.84E+04
MEDN1677	Adenosine 2′-phosphate	[M−H]^−^	C_10_H_14_N_5_O_7_P	7.06E+06	5.14E+05	4.56E+06	5.73E+05	1.79E+06	2.15E+05	1.50E+06	2.66E+05
MEDP0146	1-Methyladenine	[M+H]^+^	C_6_H_7_N_5_	5.63E+04	3.89E+04	9.63E+04	2.60E+04	6.90E+05	8.01E+04	3.54E+05	2.59E+04
MEDP0150	2′-Deoxyinosine	[M+H]^+^	C_10_H_12_N_4_O_4_	1.59E+05	2.09E+04	1.31E+06	3.45E+04	8.35E+07	2.14E+06	1.11E+06	2.99E+04
MEDP0151	2-Hydroxy-6-aminopurine	[M+H]^+^	C_5_H_5_N_5_O	2.19E+06	5.93E+04	9.77E+06	1.06E+06	2.97E+06	4.78E+05	5.15E+06	8.22E+05
MEDP0152	3′-Aenylic acid	[M+H]^+^	C_10_H_14_N_5_O_7_P	2.13E+08	3.75E+07	1.60E+07	1.77E+06	5.72E+07	1.48E+06	5.99E+06	3.77E+05
MEDP0153	3-Methylxanthine	[M+H]^+^	C_6_H_6_N_4_O_2_	4.23E+06	2.58E+05	9	0	8.67E+04	5.89E+03	9	0
MEDP0155	5-Methylcytosine	[M+H]^+^	C_5_H_7_N_3_O	8.99E+04	1.54E+04	2.19E+06	1.63E+05	6.57E+05	4.73E+04	2.27E+05	4.51E+04
MEDP0156	5-Methyluridine	[M+H]^+^	C_10_H_14_N_2_O_6_	1.92E+05	3.54E+04	5.18E+05	5.62E+04	1.97E+05	2.26E+04	3.28E+05	5.24E+04
MEDP0160	Adenosine	[M+H]^+^	C_10_H_13_N_5_O_4_	5.95E+08	1.76E+07	5.79E+08	2.33E+07	4.85E+08	1.68E+07	5.91E+08	1.71E+07
MEDP0163	Cytidine	[M+H]^+^	C_9_H_13_N_3_O_5_	6.56E+06	1.40E+06	5.16E+06	5.79E+05	7.13E+06	7.94E+05	1.50E+07	8.27E+05
MEDP0164	Cytidine-5-monophosphate	[M+H]^+^	C_9_H_14_N_3_O_8_P	2.79E+06	5.16E+05	5.26E+05	1.78E+05	1.02E+06	3.86E+05	7.86E+05	2.22E+05
MEDP0166	Deoxyguanosine	[M+H]^+^	C_10_H_13_N_5_O_4_	5.95E+08	1.76E+07	5.79E+08	2.33E+07	4.85E+08	1.68E+07	5.91E+08	1.71E+07
MEDP0168	Guanosine 3′,5′-cyclic monophosphate	[M+H]^+^	C_10_H_12_N_5_O_7_P	1.90E+05	8.07E+03	1.93E+06	6.61E+04	6.97E+05	5.16E+04	1.62E+06	6.33E+05
MEDP0169	Guanosine-5′-monophosphate	[M+H]^+^	C_10_H_14_N_5_O_8_P	7.72E+06	1.88E+06	2.50E+06	1.90E+05	1.60E+06	1.15E+05	1.64E+06	2.60E+05
MEDP0170	Hypoxanthine	[M+H]^+^	C_5_H_4_N_4_O	2.80E+06	4.04E+05	2.40E+06	5.22E+05	1.42E+06	1.04E+05	8.39E+06	9.44E+05
MEDP0177	Thymine	[M+H]^+^	C_5_H_6_N_2_O_2_	5.60E+05	2.31E+04	5.83E+05	2.77E+04	1.75E+06	2.05E+05	1.07E+06	6.06E+04
MEDP0180	Β-Nicotinamide mononucleotide	[M+H]^+^	C_11_H_15_N_2_O_8_P	4.04E+04	1.20E+04	9	0	2.24E+05	4.94E+04	9	0
MEDP0374	Cyclic Amp	[M+H]^+^	C_10_H_12_N_5_O_6_P	1.81E+06	3.39E+04	1.09E+06	5.73E+04	1.15E+07	4.60E+05	1.06E+06	3.85E+04
MEDP0383	Β-Pseudouridine	[M+H]^+^	C_9_H_12_N_2_O_6_	1.08E+06	9.66E+04	1.12E+06	5.50E+04	1.23E+06	2.15E+05	9.32E+05	6.88E+04
MEDP0390	Hypoxanthine-9-β-d-arabinofuranoside	[M+H]^+^	C_10_H_12_N_4_O_5_	2.48E+08	1.31E+07	2.03E+08	4.83E+06	1.29E+08	5.55E+06	1.95E+08	7.98E+06
MEDP0401	5′-Deoxy-5′-(methylthio) adenosine	[M+H]^+^	C_11_H_15_N_5_O_3_S	6.52E+07	3.59E+06	6.35E+06	9.74E+05	3.69E+08	9.95E+06	5.27E+06	5.96E+05
MEDP0403	Deoxycytidine	[M+H]^+^	C_9_H_13_N_3_O_4_	7.95E+04	5.63E+03	3.15E+05	2.39E+04	3.90E+05	5.68E+04	4.28E+05	6.76E+04
MEDP0526	6-Dimethylaminopurine	[M+H]^+^	C_7_H_9_N_5_	1.69E+06	1.67E+05	4.06E+06	5.36E+05	1.09E+06	5.74E+04	2.18E+07	8.70E+05
MEDP0881	Phosphocholine	[M+H]^+^	C_5_H_15_NO_4_P	2.70E+07	4.10E+06	2.54E+07	2.72E+06	3.05E+07	1.07E+06	2.05E+07	9.83E+04
MEDP0899	3-Methyladenine	[M+H]^+^	C_6_H_7_N_5_	5.63E+04	3.89E+04	9.63E+04	2.60E+04	6.90E+05	8.01E+04	3.54E+05	2.59E+04
MEDP1010	Coenzyme II (β-NADP)	[M+H]^+^	C_21_H_28_N_7_O_17_P_3_	9	0	6.64E+04	8.82E+03	2.14E+04	9.00E+03	9	0
MEDP1018	5′-Deoxyadenosine	[M+H]^+^	C_10_H_13_N_5_O_3_	4.09E+05	2.38E+04	3.72E+06	5.86E+04	2.54E+08	4.73E+05	3.14E+06	7.92E+04
MEDP1072	7-Methylguanosine	[M+H]^+^	C_11_H_15_N_5_O_5_	2.53E+05	2.03E+04	5.41E+06	3.27E+05	3.76E+06	1.42E+05	8.82E+06	1.31E+05
MEDP1073	6-O-methylguanine	[M+H]^+^	C_6_H_7_N_5_O	7.39E+05	1.60E+05	1.92E+07	9.05E+05	8.41E+05	1.83E+05	1.96E+06	8.30E+04
MEDP1077	2′-O-methyladenosine	[M+H]^+^	C_11_H_15_N_5_O_4_	1.84E+08	3.82E+07	1.31E+07	1.34E+06	5.43E+07	1.29E+06	5.28E+06	1.23E+06
MEDP1083	Oxypurinol	[M+H]^+^	C_5_H_4_N_4_O_2_	1.42E+07	6.07E+05	3.21E+07	1.71E+06	2.47E+07	3.31E+05	3.56E+07	2.89E+06
MEDP1093	Allopurinol	[M+H]^+^	C_5_H_4_N_4_O	9	0	1.95E+07	1.09E+06	6.70E+06	4.55E+05	1.45E+07	9.22E+05
MEDP1100	2-Aminomethylpyrimidine	[M+H]^+^	C_5_H_7_N_3_	1.46E+07	1.49E+06	1.00E+07	1.13E+06	1.55E+07	2.02E+05	1.39E+06	1.06E+05
MEDP1193	Flavin single nucleotide (FMN)	[M+H]^+^	C_17_H_21_N_4_O_9_P	5.39E+06	5.00E+05	2.44E+06	1.02E+05	1.58E+06	1.34E+05	1.88E+06	1.50E+05
MEDP1202	5′-Deoxy-5′-fluoroadenosine	[M+H]^+^	C_10_H_12_FN_5_O_3_	3.90E+07	2.01E+06	3.17E+07	1.44E+06	2.14E+07	9.14E+05	3.03E+07	1.00E+06
MEDP1276	Guanine	[M+H]^+^	C_5_H_5_N_5_O	2.19E+06	5.93E+04	9.77E+06	1.06E+06	2.97E+06	4.78E+05	5.15E+06	8.22E+05
MEDP1295	Cytarabine	[M+H]^+^	C_9_H_13_N_3_O_5_	1.44E+06	1.76E+05	2.34E+06	1.89E+05	2.47E+06	1.92E+05	5.05E+06	6.97E+05
MEDP1493	N6-methyladenosine	[M+H]^+^	C_11_H_15_N_5_O_4_	7.30E+05	6.74E+04	8.53E+06	1.42E+05	1.23E+06	4.02E+04	2.22E+07	1.43E+06
MEDP1498	8-Azaguanine	[M+H]^+^	C_4_H_4_N_6_O	9.44E+06	5.57E+05	2.21E+07	4.92E+05	1.64E+07	8.26E+05	2.49E+07	1.87E+06
MEDP1501	Isocytosine	[M+H]^+^	C_4_H_5_N_3_O	1.60E+06	9.18E+04	1.50E+06	1.82E+05	2.07E+06	5.34E+05	2.94E+06	3.46E+05
MEDP1654	1-Methyladenosine	[M+H]^+^	C_11_H_15_N_5_O_4_	7.30E+05	6.74E+04	8.53E+06	1.42E+05	1.23E+06	4.02E+04	2.22E+07	1.43E+06
MEDP1712	1-Methylguanosine	[M+H]^+^	C_11_H_15_N_5_O_5_	2.75E+05	1.08E+04	5.98E+06	4.08E+05	3.96E+06	3.07E+04	8.95E+06	1.72E+05
MEDP1713	1-Methylinosine	[M+H]^+^	C_11_H_14_N_4_O_5_	9	0	5.33E+05	1.00E+05	1.91E+05	1.48E+04	2.88E+05	5.39E+04
MEDP1714	2′-Deoxyadenosine	[M+H]^+^	C_10_H_15_N_5_O_4_	1.06E+05	3.21E+04	2.09E+06	3.63E+05	2.84E+05	4.88E+04	2.90E+05	4.97E+04
MEDP1717	2′-O-methylcytidine	[M+H]^+^	C_10_H_15_N_3_O_5_	1.06E+06	1.14E+05	2.94E+06	1.13E+05	3.56E+07	2.40E+06	1.44E+06	2.21E+05
MEDP1719	2′-O-methylguanosine	[M+H]^+^	C_11_H_15_N_5_O_5_	2.75E+05	1.08E+04	5.98E+06	4.08E+05	3.96E+06	3.07E+04	8.95E+06	1.72E+05
MEDP1736	5-Methylcytidine	[M+H]^+^	C_10_H_15_N_3_O_5_	1.06E+05	1.57E+04	7.53E+06	2.53E+06	3.57E+04	6.65E+03	2.11E+05	3.41E+04
MEDP1787	N4-Acetylcytidine	[M+H]^+^	C_11_H_15_N_3_O_6_	4.50E+05	6.90E+04	1.35E+06	3.31E+04	2.04E+06	3.38E+04	6.57E+05	1.04E+05
MEDP1788	N4-Acetylcytidine triphosphate	[M+H]^+^	C_11_H_18_N_3_O_15_P_3_	1.21E+07	3.84E+05	4.12E+05	8.99E+04	1.21E+07	7.63E+05	1.07E+07	5.67E+05
MEDP1791	Nicotinamide riboside (chloride)	[M-Cl]^+^	C_11_H_15_ClN_2_O_5_	5.97E+04	1.22E+04	1.43E+05	2.17E+04	5.42E+05	7.44E+04	9.00E+00	0.00E+00
MEDP1949	5-Aminoimidazole ribonucleotide	[M+H]^+^	C_8_H_14_N_3_O_7_P	1.73E+07	3.34E+06	5.13E+06	3.33E+05	1.35E+07	2.58E+06	3.03E+06	2.92E+05
MEDP1950	2′-Deoxyinosine-5′-monophosphate	[M+H]^+^	C_10_H_13_N_4_O_7_P	1.65E+07	2.69E+06	2.42E+05	7.46E+04	1.65E+05	8.50E+03	1.06E+07	9.36E+05
MEDP1954	N6-(2-Hydroxyethyl)adenosine	[M+H]^+^	C_12_H_17_N_5_O_5_	1.39E+05	2.94E+04	2.35E+08	1.20E+07	2.18E+08	9.01E+06	1.58E+06	8.41E+04
MEDP1955	3′-Deoxyadenosine	[M+H]^+^	C_10_H_13_N_5_O_3_	4.09E+05	2.38E+04	3.72E+06	5.86E+04	2.54E+08	4.73E+05	3.14E+06	7.92E+04
MEDP1956	2-Aminopurine	[M+H]^+^	C_5_H_5_N_5_	6.13E+06	2.76E+05	4.93E+06	3.16E+05	2.43E+06	1.79E+05	4.79E+06	2.31E+05
MEDP1959	N6-(4-hydroxybenzyl)adnine riboside	[M+H]^+^	C_12_H_11_N_5_O	9	0	3.07E+05	9.23E+04	1.41E+06	2.05E+05	9	0
MEDP1960	Isopentenyladenine-7-N-glucoside	[M+H]^+^	C_16_H_23_N_5_O_5_	1.13E+06	4.08E+05	1.32E+06	2.34E+05	2.18E+06	2.66E+05	9.42E+05	1.17E+05
MEDP1961	Ribosyladenosine	[M+H]^+^	C_15_H_21_N_5_O_8_	5.90E+05	8.22E+04	2.39E+06	1.17E+05	9.02E+06	7.88E+05	1.94E+07	1.09E+06
MEDP2049	N-Benzoyl-2′-deoxycytidine	[M+H]^+^	C_16_H_17_N_3_O_5_	1.56E+05	8.90E+03	1.54E+05	1.44E+04	1.81E+05	4.25E+04	1.84E+05	5.96E+03
MEDP2053	Xanthosine-5′-monophosphate	[M+H]^+^	C_10_H_13_N_4_O_9_P	7.12E+05	2.15E+05	2.46E+05	3.39E+04	1.46E+05	5.87E+04	1.14E+05	2.06E+04

### Ecological niche modelling of *Cordyceps* species

The optimized parameters were used to simulate the suitable growth area of *C. sinensis, C. cicadae, C. militaris* and *C. gunnii.* The optimized parameters of *C. sinensis* were FC = LQ and RM = 0.1. The optimized parameters of *C. cicadae* were FC = QP and RM = 0.7; the optimized parameters of *C. militaris* were FC = LQ and RM = 0.2. The optimized parameters of *C. gunnii* were FC = P and RM = 2.3. When the optimization parameters (FC and RM values) were used to set the model, the delta AICc values were all 0 (Supplementary Table 1S). The weight of each ecological factor in the habitat suitability was analysed by the jackknife method in the MaxEnt model. The percentage contribution values of each bioclimatic variable to *C. sinensis, C. cicadae, C. militaris and C. gunnii* are shown in Table 2. All values are averages of 10 repetitions. The top three contributing variables of *C. sinensis* were the mean temperature of warmest quarter (bio10), the temperature seasonality (standard deviation × 100) (bio4) and the annual mean temperature (bio1), and the relative contribution degrees were 27.86%, 26.33% and 19.39%, respectively. The top three contributioning variables of *C. cicadae* were the min temperature of coldest month (bio6), precipitation of coldest quarter (bio19) and isothermality (bio3), with relative contributions of 75.09%, 4.74% and 4.71%, respectively. The top three contributions of *C. militaris* climate variables were annual precipitation (bio12), temperature seasonality (standard deviation × 100) (bio4), and precipitation of warmest quarter (bio18), and their relative contributions were 44.51%, 13.23% and 12.09%, respectively. The top three contributions of *C. gunnii* climate variables were the min temperature of coldest month (bio6), temperature seasonality (standard deviation × 100) (bio4) and the mean temperature of coldest quarter (bio11), with relative contribution rates of 33.37%, 30.77% and 19.21%, respectively.

**Table 2:** Ecological climatic variables and niche model contribution of *C.sinensis, C.cicadae, C.militaris* and *C.gunnii.*

	Climatic variables	Contribution (%)	Permutation importance (%)	Bioclimatic suitable range	Description
*C. sinensis*	BIO1	19.394	15.109	> − 2.896, < 3.820	Annual mean temperature (°C)
	BIO3	1.576	0.149	> 36.807, < 43.624	Isothermality (BIO2/BIO7) (×100)
	BIO4	26.332	32.904	> 625.292, < 802.647	Temperature seasonality (standard deviation × 100) (C of V)
	BIO5	1.791	2.003	> 15.284, < 20.769	Max temperature of warmest month (°C)
	BIO6	0.705	3.875	> − 18.382, < − 8.802	Min temperature of coldest month (°C)
	BIO8	0.569	2.154	> 6.975, < 13.119	Mean temperature of wettest quarter (°C)
	BIO10	27.864	7.760	> 7.402, < 13.238	Mean temperature of warmest quarter (°C)
	BIO11	10.068	10.810	> − 11.700, < − 3.695	Mean temperature of coldest quarter (°C)
	BIO12	1.648	4.939	> 469.623, < 786.046	Annual precipitation (mm)
	BIO14	1.623	0.862	> 1.934, < 6.845	Precipitation of driest month (mm)
	BIO16	1.070	8.580	> 253.864, < 389.728	Precipitation of wettest quarter (mm)
	BIO18	4.929	10.522	> 245.872, < 384.400	Precipitation of warmest quarter (mm)
	BIO19	2.431	0.332	> 7.056, < 32.828	Precipitation of Coldest Quarter (mm)
*C. cicadae*	BIO1	0.273	0.190	< − 15.125, > 15.161	Annual mean temperature (℃)
	BIO3	4.708	1.683	> 34.068	Isothermality (bio2/bio7) (×100)
	BIO4	4.490	5.587	< 767.829	Temperature seasonality (standard deviation × 100) (C of V)
	BIO5	0.462	0.037	> 29.287	Max temperature of warmest month (°C)
	BIO6	75.088	57.472	> − 3.863, < 3.858	Min temperature of coldest month (°C)
	BIO8	1.650	0.740	< − 21.296, > 21.242	Mean temperature of wettest quarter (°C)
	BIO10	0.395	0.162	> 23.719	Mean temperature of warmest quarter (°C)
	BIO11	0.595	0.823	> − 6.854, < 6.860	Mean temperature of coldest quarter (°C)
	BIO12	0.244	0.013	> 1250.226	Annual precipitation (mm)
	BIO14	2.017	1.740	> 28.762	Precipitation of driest month (mm)
	BIO16	4.103	6.870	> 546.112	Precipitation of wettest quarter (mm)
	BIO18	1.233	3.765	> 496.156	Precipitation of warmest quarter (mm)
	BIO19	4.743	20.918	> 130.896	Precipitation of coldest quarter (mm)
*C. militaris*	BIO1	1.127	2.763	> 14.928	Annual mean temperature (℃)
	BIO3	1.499	0.365	> 39.130	Isothermality(bio2/bio7) (×100)
	BIO4	12.093	23.690	< 0.501	Temperature seasonality (standard deviation × 100) (C of V)
	BIO5	4.721	29.797	> 16.389, < 27.578	Max temperature of warmest month (°C)
	BIO6	1.849	1.416	> 2.394	Min temperature of coldest month (°C)
	BIO8	5.077	25.712	> 18.413, < 20.245	Mean temperature of wettest quarter (°C)
	BIO10	1.534	1.558	> 39.130	Mean temperature of warmest quarter (°C)
	BIO11	1.368	0.024	> 8.005	Mean temperature of coldest quarter (°C)
	BIO12	44.513	9.106	> 2032.902	Annual precipitation (mm)
	BIO14	2.713	0.861	> 33.678	Precipitation of driest month (mm)
	BIO16	8.372	1.130	> 924.468	Precipitation of wettest quarter (mm)
	BIO18	13.230	0.443	> 887.079	Precipitation of warmest quarter (mm)
	BIO19	1.906	3.135	> 154.160	Precipitation of coldest quarter (mm)
*C. gunnii*	BIO1	0.359	0	> 13.644	Annual mean temperature (℃)
	BIO3	10.857	16.481	< 27.798	Isothermality (bio2/bio7) (×100)
	BIO4	30.768	28.385	< 782.786	Temperature seasonality (standard deviation × 100) (C of V)
	BIO5	0.666	0.384	> 27.860	Max temperature of warmest month (°C)
	BIO6	33.373	38.367	> − 1.254	Min temperature of coldest month (°C)
	BIO8	0.419	2.660	> 20.431	Mean temperature of wettest quarter (°C)
	BIO10	0.430	0.069	> 22.618	Mean temperature of warmest quarter (°C)
	BIO11	19.207	13.050	> 4.1866	Mean temperature of coldest quarter (°C)
	BIO12	3.146	0	> 1164.830	Annual precipitation (mm)
	BIO14	0.410	0	> 21.729	Precipitation of driest month (mm)
	BIO16	0.240	0.501	> 546.750	Precipitation of wettest quarter (mm)
	BIO18	0.061	0	> 485.224	Precipitation of warmest quarter (mm)
	BIO19	0.063	0.104	> 80.997	Precipitation of coldest quarter (mm)

The variable response curve shows how each environmental variable affects the MaxEnt prediction, indicating how it changes with each environmental variable. According to the response curve of environmental variables to the presence probability in the MaxEnt model (Supplementary Fig. S5), and using a presence probability greater than 0.5 as the selection condition of suitable area for *Cordyceps*, the threshold values of the dominant environmental variables affecting the distribution of suitable area for *Cordyceps* are as follows. The ranges of bio10, bio4 and bio1 are 7.42–13.24 °C, 625.29–802.65 °C and 2.90–3.82 °C, respectively, among the top three climate variables contributing to *C. sinensis*. The range of bio6, bio19 and bio3 is between − 3.86 and 3.86 °C, greater than 130.90 mm, and greater than 34.07, among the top three climate variables contributing to *C. cicadae*. The ranges of bio12, bio18 and bio4 are greater than − 1250.23 mm, 496.16 mm and less than 767.83, respectively, among the top three climate variables contributing to *C. militaris*. The ranges of bio6, bio4 and bio11 in the top three climate variables of *C. gunnii* contribution is greater than − 1.25 °C, less than 782.79 and greater than 4.19 °C, respectively.

**Figure 7:**
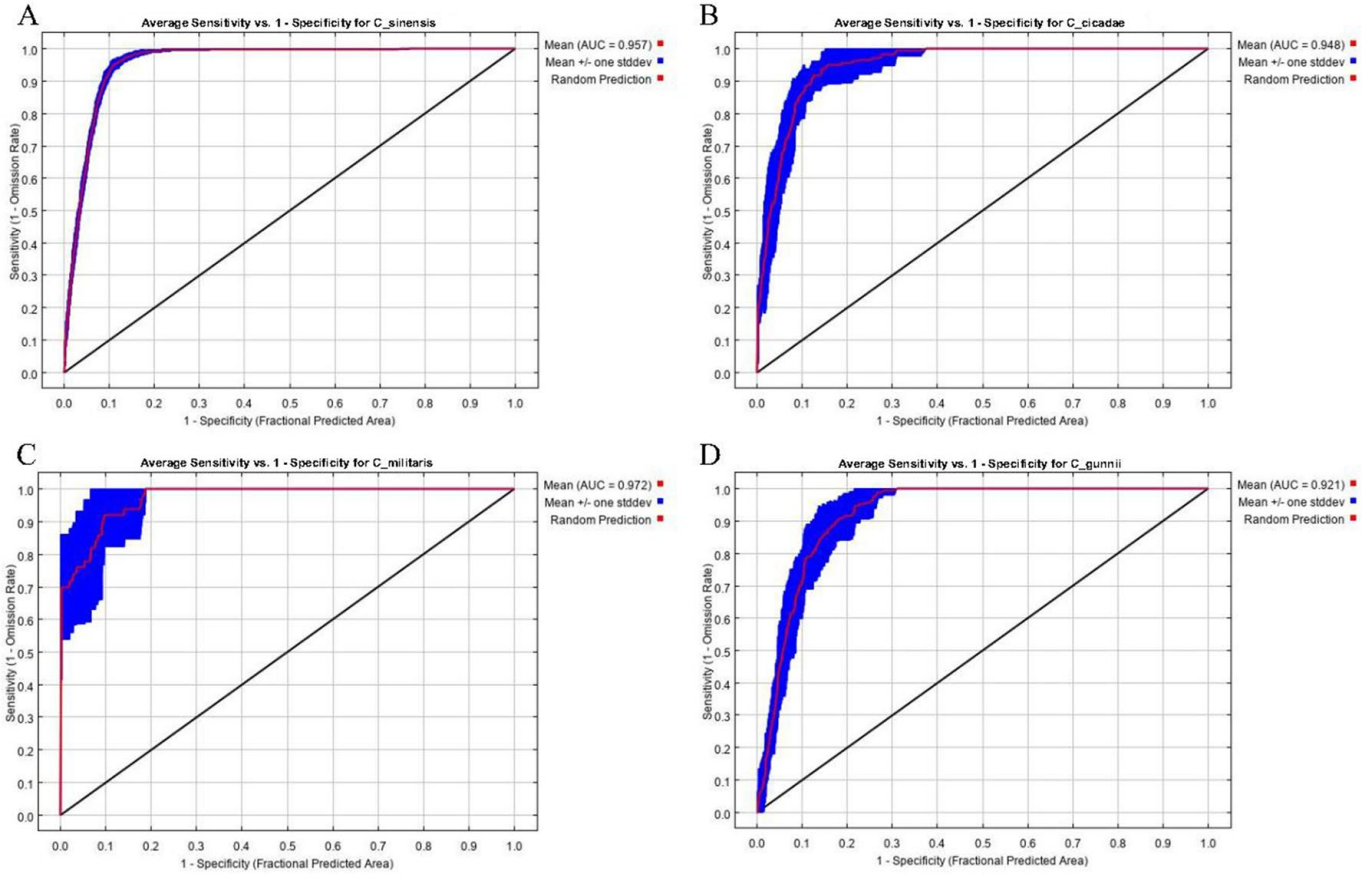
ROC curve of MaxEnt model of *Cordyceps*. (**A**) ROC curve of MaxEnt model of *C. sinensis*. (**B**) ROC curve of MaxEnt model of *C. cicadae*. (**C**) ROC curve of MaxEnt model of *C. militaris*. (**D**) ROC curve of MaxEnt model of *C. gunnii*.

The ROC curve of MaxEnt of cordyceps is shown in Fig. 7. The average AUC values of *C. sinensis, C. cicadae, C. militaris and C. gunnii* are 0.957, 0.948, 0.972 and 0.921, respectively. The results showed that the model had high reliability and accuracy in predicting the habitat suitability of *Cordyceps*.

**Figure 8:**
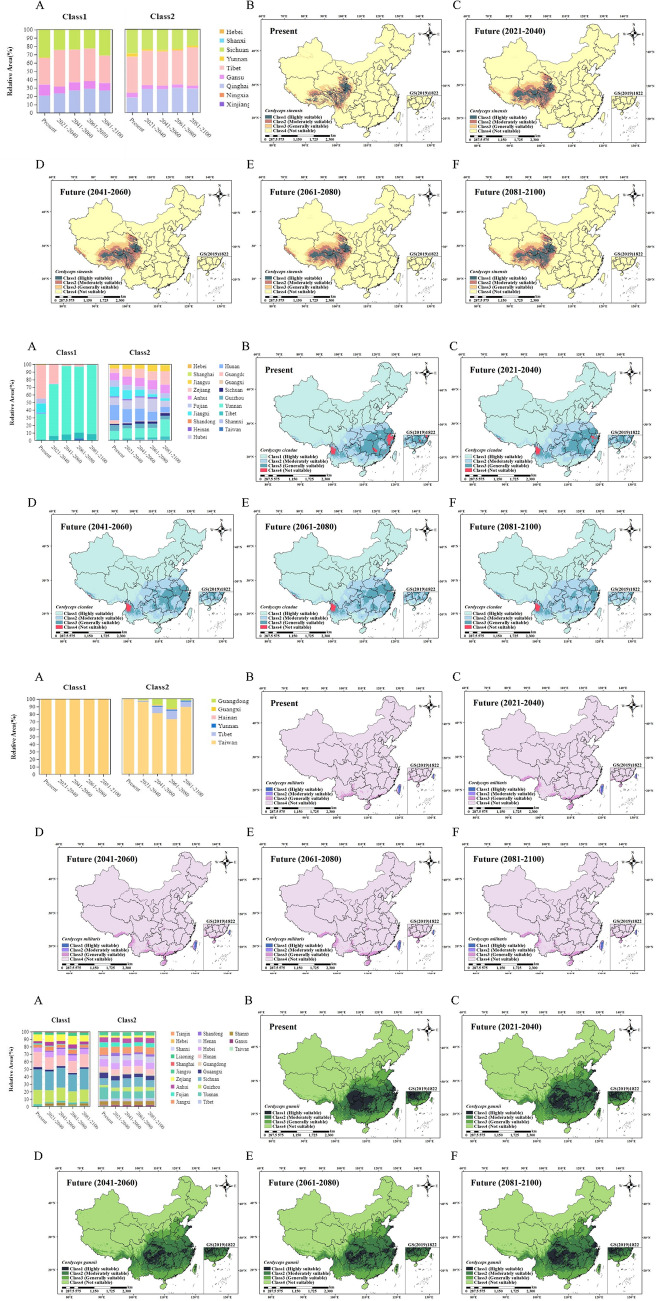
(**A**) Current situation and potential prediction of spatial suitability distribution of *C. sinensis* in China. (a) Histogram of urban area proportion in high (Class1) and medium (Class2) suitable distribution areas of *C. sinensis* in China at present. (b) Current map of suitable distribution areas of *C. sinensis* in China. (c–f) Map of suitable distribution areas of *C. sinensis* in China from 2021 to 2040, 2041 to 2060, 2061 to 2080 and 2081 to 2100. (**B**) Current situation and potential prediction of spatial suitability distribution of *C. cicadae* in China. (a) Histogram of urban area proportion in high (Class1) and medium (Class2) suitable distribution areas of *C. cicadae* in China at present. (b) Current map of suitable distribution areas of *C. cicadae* in China. (c–f) Map of suitable distribution areas of *C. cicadae* in China from 2021 to 2040, 2041 to 2060, 2061 to 2080 and 2081 to 2100. (**C**) Current situation and potential prediction of spatial suitability distribution of *C. militaris* in China. (a) Histogram of urban area proportion in high (Class1) and medium (Class2) suitable distribution areas of *C. militaris* in China at present. (b) Current map of suitable distribution areas of *C. militaris* in China. (c–f) Map of suitable distribution areas of *C. militaris* in China from 2021 to 2040, 2041 to 2060, 2061 to 2080 and 2081 to 2100. (D) Current situation and potential prediction of spatial suitability distribution of *C. gunnii* in China. (a) Histogram of urban area proportion in high (Class1) and medium (Class2) suitable distribution areas of *C. gunnii* in China at present. (b) Current map of suitable distribution areas of *C. gunnii* in China. (c–f) Map of suitable distribution areas of *C. gunnii* in China from 2021 to 2040, 2041 to 2060, 2061 to 2080 and 2081 to 2100. The figure was accomplished by ArcGIS (version 10.7, https://www.esri.com/zh-cn/arcgis/) and MaxEnt software (version 3.4.1, https://biodiversityinformatics.amnh.org/open_source/maxent/).

Figure 8A–D shows the potential distribution of *C. sinensis, C. cicadae, C. militaris and C. gunnii*. The MaxEnt models based on current climate conditions and the logistic output (LO) results generated using MaxEnt software are expressed as probabilities ranging from 0 to 1. Using the reclassification tool of ArcMap 10.7, the simulation results were divided into four levels, where 0–0.2 was considered unsuitable, 0.2–0.45 was considered low, 0.45–0.7 was considered medium, and 0.7–1 was considered high.

At present, China’s cities with high and medium suitability for *C. sinensis* (Fig. 8A) are mainly distributed in Sichuan, Tibet, Qinghai, Gansu, Yunnan, covering an area of 327,218 km^2^, accounting for 88.94% of the total area of *C. sinensis*. Cities with high and medium suitability (Fig. 8B) for *C. cicadae* are mainly distributed in Yunnan, Jiangxi, Xizang, Fujian and Guizhou, covering an area of 274,115 km^2^, accounting for 74.51% of the total area of *C. cicadae*. Cities with high and medium suitability for *C. militaris* (Fig. 8C) are mainly distributed in Guangxi, Xizang, Yunnan, Taiwan, Guangdong, covering an area of 360,226 km^2^, accounting for 97.91% of the total area of *C. militaris*. Cities with high and medium suitability for *C. gunnii* (Fig. 8D) are mainly distributed in Guizhou, Sichuan, Guangxi, Anhui, Hubei, covering an area of 238,971 km^2^, accounting for 64.96% of the total area of *C. gunnii*. According to the future prediction, the suitability distribution area of *C. cicadae* showed a significant decline. However, *Cordyceps sinensis* showed a significant increase in its suitability distribution region during 2021–2040 and a small increase in its suitability region during 2041–2060, 2061–2080 and 2080–2100, at which time it was basically stable. In the three periods of 2021–2040, 2041–2060 and 2061–2080, the suitable area of *C. militaris* increased, and in the period of 2080–2100, the area of suitable area decreased sharply. From 2021 to 2040, the suitable area of *C. gunnii* was in the same state as that of *C. sinensis*: the area initially increased, then decreased from 2041 to 2060, and finally slowly increased from 2041 to 2100. In addition, Table 3 shows that in the four future periods (2021–2040, 2041–2060, 2061–2080, 2081–2100), the centroid shifts distances of *C. sinensis* are 141.32, 119.06, 135.63 and 123.91 km, the migration distances of *C. militaris* are 367.43, 371.14, 343.71 and 376.60 km, the migration distances of *C. cicadae* are 76.52, 78.22, 157.38 and 80.15 km, and the migration distances of *C. gunnii* are 75.84, 77.28, 69.61 and 77.73 km, respectively. *Cordyceps sinensis, C. cicadae, C. militaris and C. gunnii* have the same direction of centroid shifts and shifted to the northwest (Supplementary Fig. 6S).

**Table 3:** Four future periods (2021–2040, 2041–2060, 2061–2080, 2081–2100) change of centroid migration in suitable areas of *C. sinensis, C. cicadae, C. militaris and C. gunnii.*

	Period	Species			
		*C. sinensis*	*C. militaris*	*C. gunnii*	*C. cicadae*
Coordinate (latitude, longitude)	Current	97.53E, 32.00N	110.31E, 23.29N	110.44E, 29.08N	110.66E, 28.61N
	1 Future2021-2040	96.11E, 32.32N	106.64E, 24.14N	110.47E, 29.76N	110.26E, 29.20N
	2 Future2041-2060	96.38E, 32.40N	106.57E, 24.05N	110.28E, 29.76N	110.33E, 29.25N
	3 Future2061-2080	96.23E, 32.48N	106.89E, 24.12N	110.19E, 29.67N	109.16E, 29.16N
	4 Future2081-2100	96.30E, 32.34N	106.57E, 24.23N	110.43E, 29.78N	110.19E, 29.20N
Distance (km)	1 Future2021-2040 (d1)	141.32 km	367.43 km	75.84 km	76.52 km
	2 Future2041-2060 (d2)	119.06 km	371.14 km	77.28 km	78.22 km
	3 Future2061-2080 (d3)	135.63 km	343.71 km	69.61 km	157.38 km
	4 Future2081-2100 (d4)	123.91 km	376.60 km	77.73 km	80.15 km

In conclusion, under future climate conditions, the area of suitable areas for *C. cicadae* will gradually decrease and will generally increase compared with the area of suitable areas for *C. sinensis, C. gunnii, C. militaris*. The suitable areas for *C. sinensis, C. cicadae, C. militaris and C. gunnii* all migrated to the northwest. According to the biological variables of 13 kinds of climatic factors, pearson correlation analysis showed (Fig. 6A) that there were strong positive correlations between various groups; for *C. cicadae and C. militaris*, there was a correlation coefficient between 0.8 and 1.0. In addition, there was a strong positive correlation between *C. sinensis* and *C. cicadae* and between *C. sinensis* and *C. gunnii*. There was a weak positive correlation between *C. gunnii* and *C. militaris* and between *C. militaris* and *C. sinensis*; the correlation coefficient was between 0.2 and 0.4. There was a very weak positive correlation between *C. sinensis* and *C. militaris*, with a correlation coefficient between 0.0 and 0.2. The results indicated that the bioclimatic environments of *C. sinensis*, *C. gunnii* and *C. cicadae* were similar.

### Correlation analysis of climate factors and metabolites

Procrustes is used to analyse the correlation between two groups of data^37^. It is a method for comparing the consistency of two groups of data. In Fig. 6B, different colours represent different groups. The points mapped on the principal orthogonal axis are the quadrate points from the climate factor PCA, represented by solid circles. The points mapped on the oblique orthogonal axis are the quadrate points from the nucleoside compound PCA, represented by triangles. The line indicates the two matched samples. The length of the line segment is the residual value between the two samples. The value of M^2^ is 0.096, which is the sum of squares of residual values. The smaller the value of M^2^ is, the better the consistency between the climate factors of *C. sinensis, C. cicadae, C. militaris*, *C. gunnii* and the data of nucleoside compounds. The significance *p value* of M^2^ calculated by the substitution test is 0.001.

The correlation coefficient and *p value* between the biomass and the relative content of all metabolites of 13 climate factors were calculated; the threshold condition of the correlation range between 0 and 1, significant correlation coefficient of 0.8 and significant correlation *p value* of 0.01 were selected to decide the results of significant correlation. Cytoscape was used to map the network. As shown in Fig. 8C, 13 climate factors were significantly correlated with 75 nucleoside metabolites, including 30 nucleoside compounds related to bio5, 27 nucleoside compounds related to bio4, 24 nucleoside compounds related to bio10, and 19 nucleoside compounds related to bio1. There were 18 nucleosides related to bio12, bio16 and bio18; 16 to bio3; 15 to bio6; 14 to bio19; and 13 to bio14. There were 8 nucleoside compounds related to bio11 and 3 nucleoside compounds related to bio8. As shown in Supplementary Fig. 3S, different compounds have different correlations with different climate factors. 3′-Deoxyadenosine (cordycepin, MEDP1955), 5′-deoxyadenosine (MEDP1018) and 2-deoxyinosine (MEDP0150) were fully correlated with bio5 (r = 1). The main nucleoside compounds uracil (MEDN0169) and bio5, bio4, 3′-deoxyadenosine (cordycepin, MEDP1955) and bio4, thymine (MEDP0177) and bio5, bio4, adenosine (MEDP0160) and bio4, bio5, Inosine (MEDN1003) were significantly correlated with bio6, and uracil (MEDN0169) was significantly correlated with bio4. The results showed that bio4 and bio5 were the most influential climatic factors on nucleoside compounds.

Pearson analysis of 9 principal components of nucleoside compounds and 13 climate factors showed that the correlation heatmap (Supplementary Fig. 4S) was significantly correlated with climate factors (*p value* < 0.05), which were adenine, thymidine, uracil, inosine, adenosine, thymine and 3′-deoxyadenosine. Combined with correlation coefficient > 0.6 were bio4 and uracil, adenosine, thymine, 3′-deoxyadenosine; bio5 and uracil, adenosine, thymine, 3′-deoxyadenosine; bio6 and inosine; bio8 and adenine, inosine, thymine; bio10 and thymidine; bio11 and uracil; bio14 and inosine; bio18 and thymidine, bio19 and inosine. According to the correlation network (Fig. 6D) analysis of 9 principal components of nucleoside compounds and 13 climatic factors, these results show consistency with the above results, and bio5 and bio4 were the most influential climatic factors on the compounds. The results showed that bio4 and bio5 played a decisive role in uracil, adenosine, thymine and 3′-deoxyadenosine, and bio6 had a significant correlation with inosine. The important roles of bio5 and bio4 in nucleoside compounds were also confirmed.

## Discussion

The quality of TCM mainly depends on the type and content of the main active chemical composition, which is affected by many factors and fluctuates. *Cordyceps* is a rare TCM that can be used as both medicine and food in China. In fact, of all available literature reporting on *Cordyceps*, *C. sinensis* and *C. militaris* accounted for 60% of the total reports. *Cordyceps militaris* has been used as a tonic in China for hundreds of years, has been reported to have similar health benefits as *C. sinensis* and is used as an alternative^38–41^. Research performed on *C. cicadae* over the past two decades has shown that it possesses biological properties and bioactive compounds similar to those of *C. sinensis* and *C. militaris* and has suggested that it can be used as an alternative source of *Cordyceps*^13^. In addition, *Cordyceps gunnii* is popularly referred to as Chinese rare caterpillar fungus^18^. Therefore, *Cordyceps cicadae*, *C. militaris* and *C. gunnii* were selected as the candidates for substitution in this study. In this study, metabolomics was used for the first time to detect and analyse all metabolites of *C. sinensis, C. cicadae, C. militaris and C. gunnii*, and the suitability distribution was predicted by an ecological niche model. The MaxEnt model was used to predict the suitable habitat areas of *Cordyceps*, and ArcGIS was used for visual analysis. Correlation analysis of chemical components and eco-climate factors was conducted to explore the effects of climate factors on the main active components of *Cordyceps*, and temperature seasonality and max temperature of the warmest month had the most prominent effects on nucleosides. The results showed that the distribution area of *C. sinensis, C. cicadae and C. gunnii* species was consistent with the recorded distribution, while *C. militaris* was predicted to be distributed in Guangdong and Taiwan, indicating that the artificial cultivation of cordyceps could be considered in these areas. In the predicted future *C.* suitability distribution region, the area of suitable areas for *C. cicadae* is generally decreasing, which may be due to the lack of artificial cultivation. Compared with *C. cicadae*, the area of suitable areas for *C. sinensis, C. militaris and C. gunnii* is generally rising gently, which may alleviate the problem of resource exhaustion, but the overall effect is not good. However, in addition to climate variables, other factors, including soil, terrain, biological factors and human activities, also affect the content of nucleoside compounds in cordyceps and are key to the formation of medicinal plant metabolites. Further studies incorporating these factors into the model will further enhance the prediction of possible changes in the distribution of *Cordyceps* in China due to changes in climatic conditions. In this study, we successfully established a distribution model of potential species of *C. sinensis, C. cicadae, C. militaris* and *C. gunnii* with changes in climate variables and estimated the impact of climate change on the suitable habitat of *Cordyceps*.

Cross-analysis of various techniques showed that *C. sinensis, C. cicadae,* and *C. gunnii* had a strong correlation of compounds and climate factors, indicating that their growing environmental conditions and nucleoside compounds were similar. It is suggested that experts consider *C. cicadae* and *C. gunnii* as new medicinal sources of *C. sinensis* and include them in the pharmacopoeia. The results provide a basis for the sustainable development of *C. sinensis* resources and offer a new way to evaluate the impact of climate factors on the quality of TCM.

## Methods

### Fungal sample materials and coordinate data sources

*Cordyceps sinensis*, *C. cicadae*, *C. militaris* and *C. gunnii* were purchased from Yunnan Province, Anhui Province, Liaoning Province and Guizhou Province, respectively, and were identified by Professor Linfang Huang, Institute of Medicinal Plants, China Academy of Chinese Medical Sciences and Peking Union Medical College. They were purchased in 2014 (Fig. 2). Low temperature storage was employed (− 20 °C). For a total of 12 samples, each sample set consisted of three biological replicates. Specific sample information is shown in Table 4.

**Table 4:** Source information of *C. sinensis*, *C. cicadae*, *C. militaris* and *C. gunnii* samples.

Code	Region	Latitude, longitude
*C. sinensis*	Yunnan Province, China	99.688, 27.813
		100.012, 27.005
		100.002, 27.001
*C. cicadae*	Anhui Province, China	116.104, 31.229
		116.103, 31.225
		116.101, 31.225
*C. militaris*	Liaoning Province, China	123.226, 42.092
		123.227, 42.088
		123.221, 42.087
*C. gunnii*	Guizhou Province, China	108.146, 27.313
		108.136, 27.027
		108.144, 27.026

The coordinate data of *C. sinensis*, *C. cicadae*, *C. militaris* and *C. gunnii* were mainly obtained from (1) the Chinese Virtual Herbarium (CVH, https://www.cvh.ac.cn/), (2) the National Specimen Information Infrastructure (NSII, http://www.nsii.org.cn/2017/home.php), (3) the Global Biodiversity Information Facility (GBIF, https://www.gbif.org/), (4) published literature, and (5) at the sampling site. The sample distribution information was quickly obtained through retrieval, totalling more than 4600 occurrences. Since the location of most sample points was not accurate, the detailed geographic location of sample collection could not be accurately expressed. We used the Trim Duplicate Occurrences tool of ENMTools V1.4.4^42^ and Baidu tools (http://api.map.baidu.com/lbsapi/getpoint/index.html) to confirm the geographic coordinates of each distribution record, eliminate specimens without location coordinates and duplicate data, and filter the species data. The spatial resolution of the bioclimatic variables was 2.5 arc-min (approximately 4.5 km^2^), and the buffer distance was set to 3 km. Only one distribution point was retained when the distance between the distribution points was less than 3 km. After duplicate points were removed, the error of the clustering effect in predicting potential distribution areas could be reduced, and the model overfitting could be reduced^43,44^. We understood the spatial autocorrelation to be due to overfitting of the data results caused by an overdense sample size in some regions^45^. These operations can greatly reduce the spatial autocorrelation of species occurrence data and effectively reduce the error^46^. Finally, data from 2529 samples of *C. sinensis*, *C. cicadae*, *C. militaris* and *C. gunnii* were obtained (Fig. 2).

### *Cordyceps* sample extraction

The sample was thawed on ice. Cold steel balls were added to the mixture and homogenized at 30 Hz for 3 min. Then, 1 mL of 70% methanol with internal standard extract was added to the homogenized centrifuge tube, and the mixture was swirled for 5 min and then centrifuged at 12,000 rpm at 4 °C for 10 min. After centrifugation, 400 µL of supernatant was drawn into the corresponding EP tube and stored in a − 20 °C refrigerator overnight, centrifuged at 12,000 r/min at 4 °C for 3 min, and 200 µL of supernatant was placed in the liner of the corresponding injection bottle for on-board analysis.

### Conditions of liquid chromatography and mass spectrometry

T3 UPLC Conditions: The sample extracts were analysed using an LC‒ESI‒MS/MS system (UPLC, ExionLC AD, https://sciex.com.cn/; MS, QTRAP^®^ System, https://sciex.com/). The analytical conditions were as follows: UPLC: column, Waters ACQUITY UPLC HSS T3 C18 (1.8 μm, 2.1 mm × 100 mm); column temperature, 40 °C; flow rate, 0.35 mL/min; injection volume, 5 µL; solvent system, water (0.1% formic acid): acetonitrile (0.1% formic acid); gradient program, 95:5 V/V at 0 min, 10:90 V/V at 10.0 min, 10:90 V/V at 11.0 min, 95:5 V/V at 11.1 min, 95:5 V/V at 14.0 min.

QTOF-MS/MS: A triple TOF mass spectrometer was used due to its ability to acquire MS/MS spectra on an information-dependent basis (IDA) during an LC/MS experiment. In this mode, the acquisition software (TripleTOF 6600, AB SCIEX) continuously evaluates the full scan survey MS data as it collects and triggers the acquisition of MS/MS spectra depending on preselected criteria. In each cycle, 12 precursor ions whose intensity was greater than 100 were chosen for fragmentation at a collision energy (CE) of 30 V (12 MS/MS events with a product ion accumulation time of 50 ms each). ESl source conditions were set as follows: ion source gas 1 as 50 psi, lon source gas 2 as 50 psi, curtain gas as 25 psi, source temperature 500 °C, and lon spray voltage floating (ISVF) 5500 V or − 4500 V in positive or negative modes, respectively (Supplementary Fig. S1A,B).

### Identification and quantification of metabolites

The metabolites were identified and quantified by Jiaxing Maiwei Metabolic Biotechnology Co., Ltd. (Jiaxing, China). The LC-QTOF-MS/MS experiment was conducted based on the self-built targeted standard database MWDB (including second-order spectrum and retention time, RT) and the MHK database (including Metlin, HMDB, KEGG database information, including secondary spectrum, RT) and MetDNA, and the multi-ion pair information and RT of the identified metabolites were extracted. A multi-reaction monitoring mode (MRM) metabolite detection multipeak diagram (Supplementary Fig. S1C,D) shows the substances that can be detected in the sample, and each different colour mass spectrum peak represents a detected metabolite. The characteristic ions of each substance are screened out by a triple quadrupole rod, and the signal intensity (CPS) of the characteristic ions is obtained in the detector. Then, the chromatographic peaks are integrated and corrected, and the peak area of each chromatographic peak represents the relative content of the corresponding substance. MRM analysis of triple quadrupole mass spectrometry was used to complete the metabolite quantification (Table 1). The filter conditions of differentially accumulated metabolites (DAMs) were as follows: |log_2_ (fold change)| ≥ 1, *p value* < 0.05, and variable importance predictive (VIP) ≥ 1. Principal component analysis (PCA) was performed on the samples to preliminarily understand the overall metabolic differences among each group and the variation degree among the samples within the group^47^. To investigate the accumulation of specific metabolites, PCA and OPLS-DA were performed using R (http://www.R-project.org/).

### Climatic factor data

WorldClim is a high spatial resolution global database of weather and climate data. These data can be used for mapping and spatial modelling. This research used WorldClim version 2.1 (https://www.worldclim.org/) 19 bioclimatic variables (bio1-bio19) as environmental monitoring factors. The historical (recent) climate data from 1970 to 2000 and environmental layers of 19 bioclimatic variables for future climate scenarios (2021–2040, 2041–2060, 2061–2080, 2080–2100) were downloaded from the WorldClim Global Climate Database (http://WorldClim.org/), spatial resolution: 2.5 arc min (approximately 4.5 km^2^)^48,49^. Bioclimatic variables are used as baselines for future scenarios and represent annual trends, seasonality, and extreme or restrictive environmental factors. Multicollinearity among bioclimatic variables easily occurs, which increases the complexity of the model and affects the corresponding relationship and contribution judgement of the model. To overcome the multicollinearity problem, this article proposes a method for using multicollinearity analysis to examine the correlation between climate factors; for the correlation of absolute value between the two factors| r | > 0.8, only one factor was selected into the model. Prior to this method, climate factors should be input into the MaxEnt model to obtain the contribution degree of each climate factor, then this data can be used as the basis for multicollinearity factor elimination; that is, for factors with strong correlation, factors with a high contribution degree should be selected into the model. According to the results of factor correlation analysis, contribution evaluation and species suitability conditions, the climate variables screened out (Table 2) included annual mean temperature (bio1), isothermality (bio3), temperature seasonality (standard deviation × 100) (bio4), max temperature of warmest month (bio5), minimum temperature of coldest month (bio6), mean temperature of wettest quarter (bio8), mean temperature of warmest quarter (bio10), mean temperature of coldest quarter (bio11), annual precipitation (bio12), precipitation of driest month (bio14), precipitation of wettest quarter (bio16), precipitation of warmest quarter (bio18) and precipitation of coldest quarter (bio19), for a total of 13.

### MaxEnt model

The algorithm of the free software MaxEnt V3.4.0 (http://bioversityinformatic.amnh.org/open_source/MaxEnt/) calculates the most likely potential geographic distribution of a species based on the relationship between geographic data and the known distribution of the target species^50^. Existing studies have shown that the MaxEnt model may not be the best model under the default setting, and the MaxEnt model is easier to overfit when the sample size is small, so it is necessary to adjust the model parameters^45,51,52^. MaxEnt software has multiple available parameter settings, and the prediction ability of statistical significance and model complexity should be considered when determining the optimal parameters. Therefore, the optimal parameter combination should be found through model calibration so that the prediction results can be as close as possible to the ideal fitness status of species^45,53–55^. In this study, we refer to the latest research progress in recent years and call an R language package called Kuenm to realize the automatic calibration and evaluation of the important parameters of the MaxEnt model. The optimal settings are selected from 600 parameter combinations, which further improves the reliability of the prediction results of suitable areas.

The two most important parameters of MaxEnt are the feature combination (FC) and regularization multiplier (RM), and the optimal selection of these two parameters contributes to significantly improving the prediction accuracy of the model^26,27,45,53,54,56^. There are 5 options for FC, which are linear (L), quadratic (Q) product (P), threshold (T) and hinge (H), and 15 different combinations are set (including L, Q, P, H, LQ, LP, LH, QP, QH, PH, LQP, LQH, LPH, QPH, LQPH). RM parameters are generally set to less than 4, and one RM value is set from 0.1 to 4 every 0.1 interval, so a total of 40 RM values are set^26^. The Kuenm program package of R software was employed, and MaxEnt was used to carry out the prediction operation of 600 different parameter models (15 FC settings and 40 RM values freely combined)^53^. This method first selects the set of models with statistically significant models and omission rates less than 5% from all the competing models, and then according to Akaike Information Criterion (AICc), selects the model with a Delta AICc value less than 2 as the recommended model^53,55,57^. If R software selects more than one recommended model, the model with the smallest Delta AICc value will be regarded as the optimal model.

Our modelling was performed according to the standard protocol for reporting species distribution models by Zurell et al.^58^. The feature parameters were set as linear features, quadratic features, product features, threshold features and hinge features, and “Create response curves”, “Make pictures of predictions” and “Do jackknife to measure variable importance” were chosen to interpret how individual variables affect the probability of the presence of Cordyceps. In the basic part, the “Random test percentage” was set as 25, representing 75% of the sample data that were randomly selected as the model training set; the remaining 25% of the sample data were used as the test set to verify the model. The “Regularization multiplier” was set as 1 to prevent overcomplexity and reduce overfitting by controlling the intensity of the chosen feature classes. The “Maximum number of background points” was set as 10,000, and the “Replicates” was set as 10. In the advanced part, the “Maximum iterations” was set as 500, and the “Convergence threshold” was set as 0.0001. The output format was set as “Cloglog”, and a previous study showed that the “Cloglog” output was the optimal output mode for predicting the suitable area^59^. The MaxEnt model used the receiver operating characteristic curve (ROC curve) analysis method to verify the accuracy of the model. The ROC curve based on the threshold relies on the judgement model precision by changing the threshold, employing the false-positive rate (the actual be predicted without the species distribution probability of positive) as the abscissa, the true positive rate (actually have the species distribution and predict positive probability) for the vertical curve, and the curve with the abscissa of area as the AUC (the value of area is between 0 and 1, which is used to measure the accuracy of the prediction results of the model). It is generally believed that AUC < 0.6 indicates that the prediction results fail, AUC > 0.8 indicates that the forecast result is good, and AUC > 0.9 is considered excellent^60^. The importance of variables was assessed using a jacknife method. Response curves were used to obtain the range of bioclimatic variables. The spatial statistical function of ArcGIS software was used to determine the suitability grades of the potential suitability areas of *C.* species according to the distribution results of the real areas of *C.* species, and the areas and proportions of different potential suitability areas could be calculated.

### Correlation analysis of climate factors and metabolites

ArcGIS was used to extract 13 key eco-climatic factors. Correlation analysis was conducted between the selected bioclimatic variables involved in the growth of *Cordyceps* and the nucleoside metabolites in *Cordyceps*. Pearson correlation coefficient (r) between climate factors and the relative contents of nucleoside compounds was calculated for correlation analysis. The screening criteria required that the correlation coefficient was in the absolute range of 0.8–1.0, indicating a strong correlation. A value of 0.6–0.8 is strongly correlated, 0.4–0.6 is moderately correlated, 0.2–0.4 is weakly correlated, and 0–0.2 is extremely weakly correlated or not correlated. Cytoscape (Cytoscape Consortium, USA, version 3.7.1) was used to visualize networks of interactions between climate factors and differential nucleoside metabolites. The R software package Draws a Pheatmap.

### Statement of licences for plant material

This research that experimental researchon cultivated plants, comply with the IUCN Policy Statement on Research Involving Species at Risk of Extinction and the Convention on the Trade in Endangered Species of Wild Fauna and Flora, and does not involve collection of wild plants.

## Supplementary Information

Supplementary Information.

## Data Availability

PCA uses the built-in statistical prcomp function of R software (http://www.r-project.org/) and sets the prcomp function parameter scale = True to normalize the data by unit variance scaling (UV scaling). HCA uses UV scaling for metabolite content data to draw heat maps through R software ComplexHeatmap package. Pearson correlation coefficients were calculated using the built-in cor function of R software. OPLS-DA uses the MetaboAnalystR package OPLSR.Anal function in R software for analysis. Procrustes analysis was performed using the OmicShare tools, a free online platform for data analysis (https://www.omicshare.com/tools). Data from the current study are available from the corresponding author upon reasonable request.

## Abbreviations

TCM: Traditional Chinese medicine
PCA: Principal component analysis
OPLS-DA: Orthogonal partial least-squares discriminant analysis
MRM: Multiple reaction monitoring
DAMs: Differentially accumulated metabolites
VIP: Variable importance predictive
