# Supplementary material for: Cordyceps cicadae and Cordyceps gunnii have closer species correlation with Cordyceps sinensis: from the perspective of metabonomic and MaxEnt models
Source: Sci Rep. 2022 Nov 28;12:20469. doi: 10.1038/s41598-022-24309-z (PMC9705360; doi:10.1038/s41598-022-24309-z)
Supplement: Supplementary file 1 — Supplementary Information. [file 41598_2022_24309_MOESM1_ESM.pdf]

Supplementary figures and tables

Supplementary figures

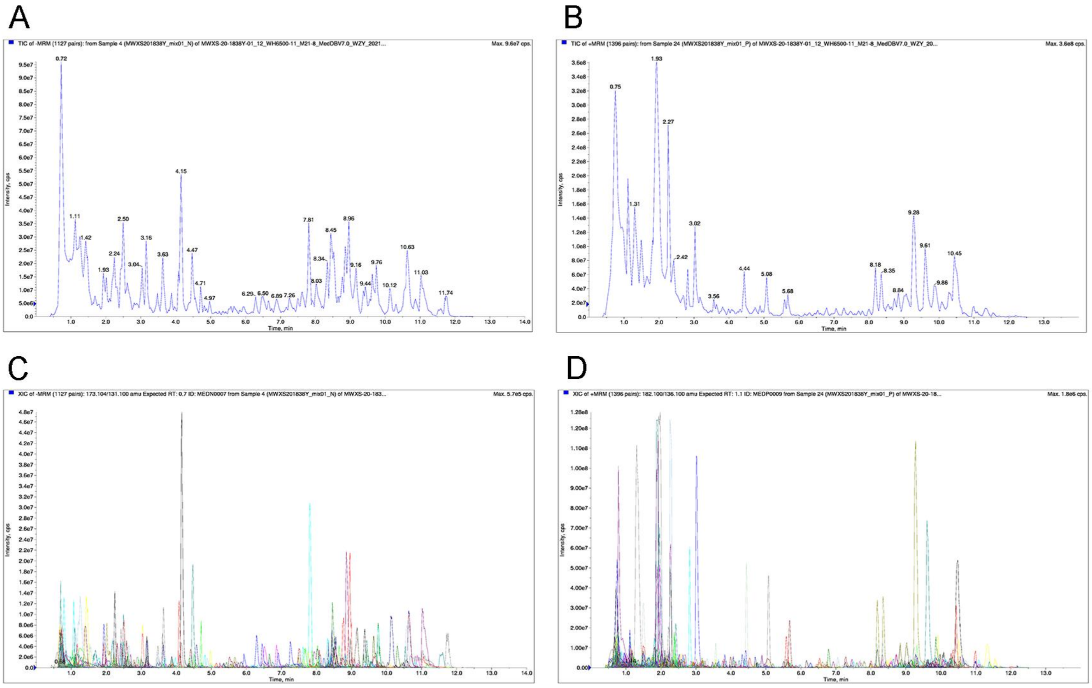

Supplementary figure S1. Total ion current diagram of the mixed sample analyzed by mass spectrometry. (A) Total ion current diagram in negative ion mode. (B) Total ion current diagram in positive mode. (C) Multi-peak diagram of MRM metabolites detection in negative ion mode. (D) Multi-peak diagram of MRM metabolites detection in negative ion mode.

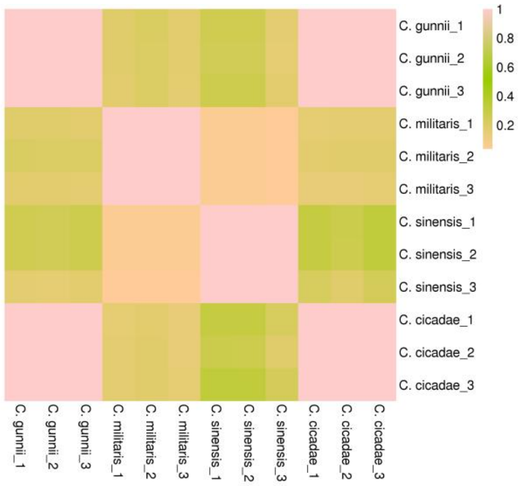

Supplementary figure S2. Correlation heat map analysis of main nucleoside components in *C.Sinensis, C. cicadae, C. militaris* and *C. gunnii.*

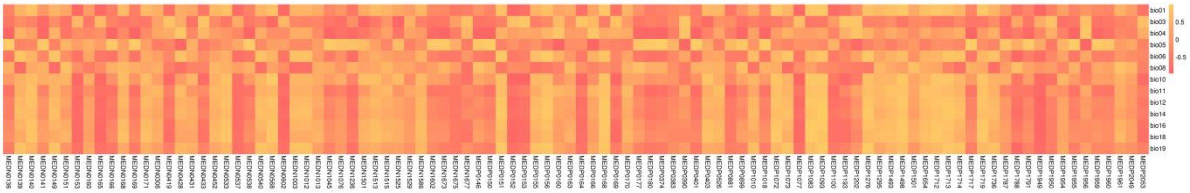

Supplementary figure S3. Pearson heat map of correlation between eco-climate factors and nucleoside metabolites.

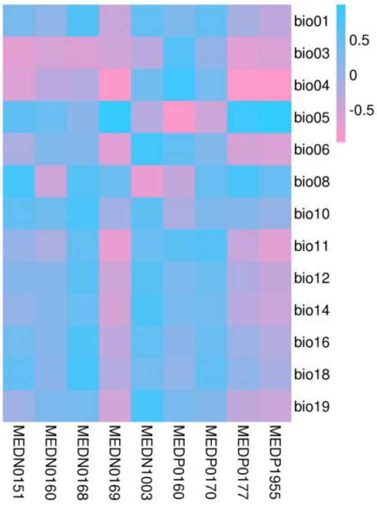

Supplementary figure S4. Pearson heat map of correlation between eco-climate factors and major components of nucleoside metabolites.

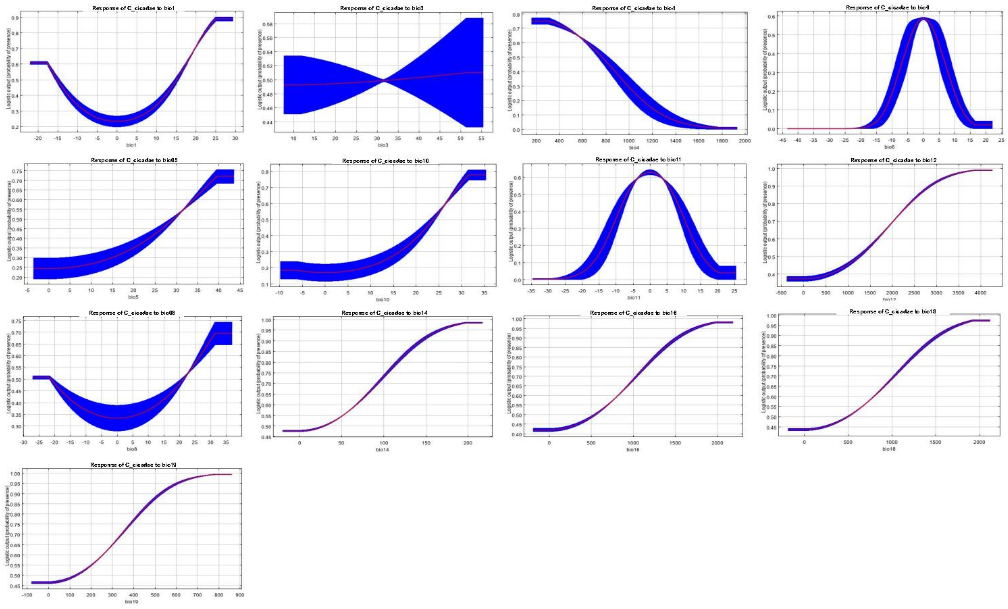

Supplementary figure S5-1. Response curves of 13 environmental variables to *C. cicadae* under current climate conditions.

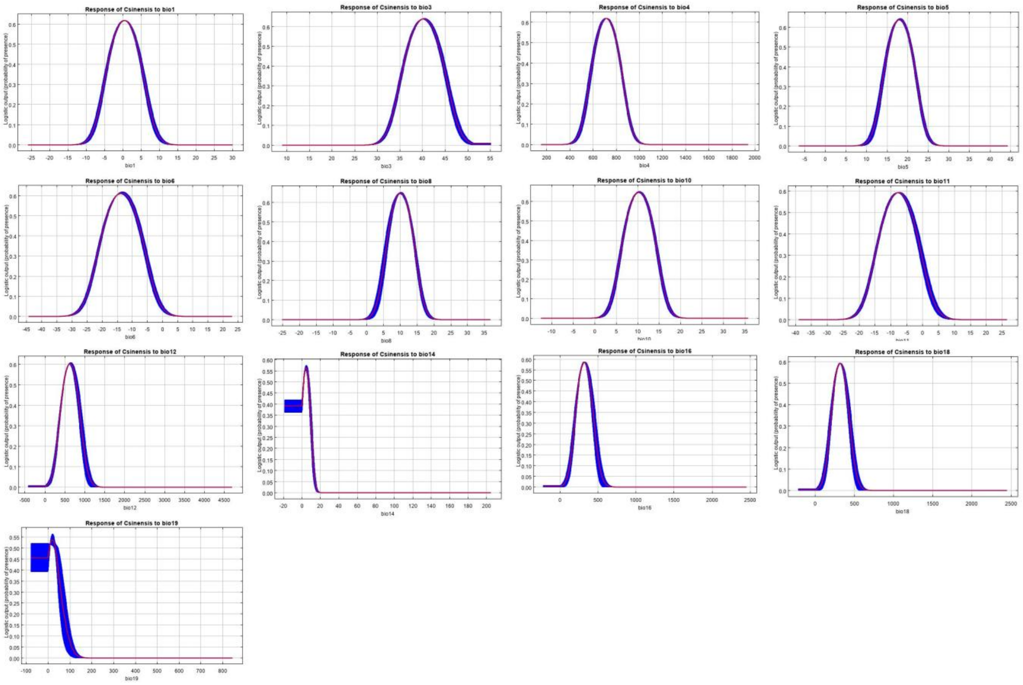

Supplementary figure S5-2. Response curves of 13 environmental variables to *C. sinensis* under current climate conditions.

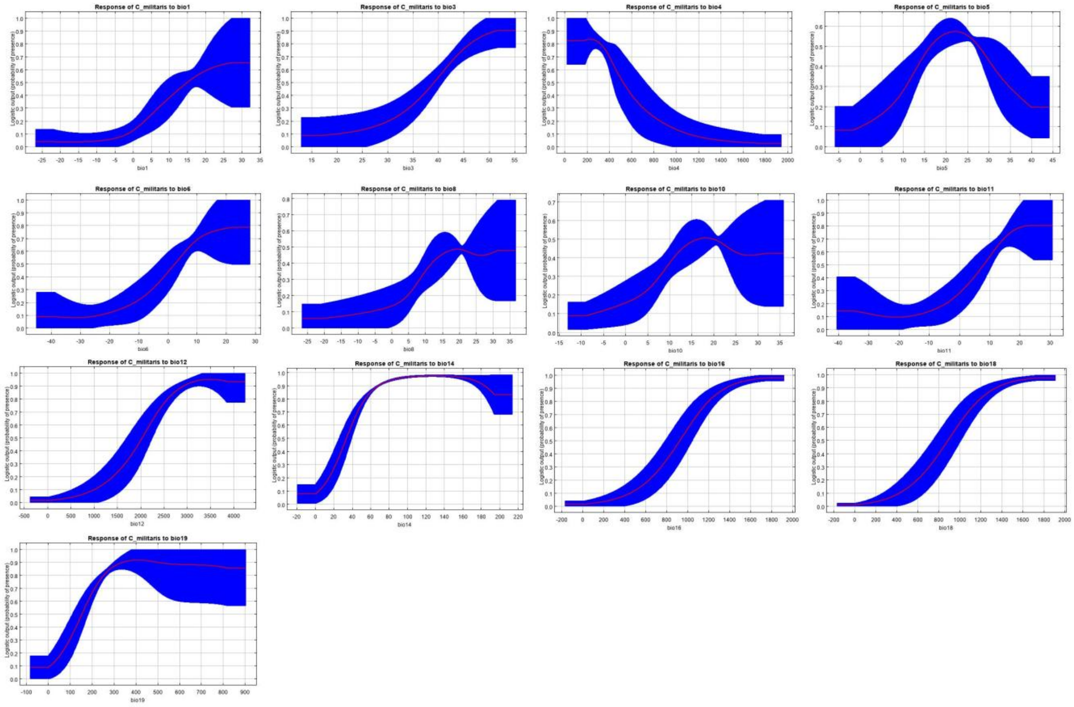

Supplementary figure S5-3. Response curves of 13 environmental variables to *C. militaris* under current climate conditions.

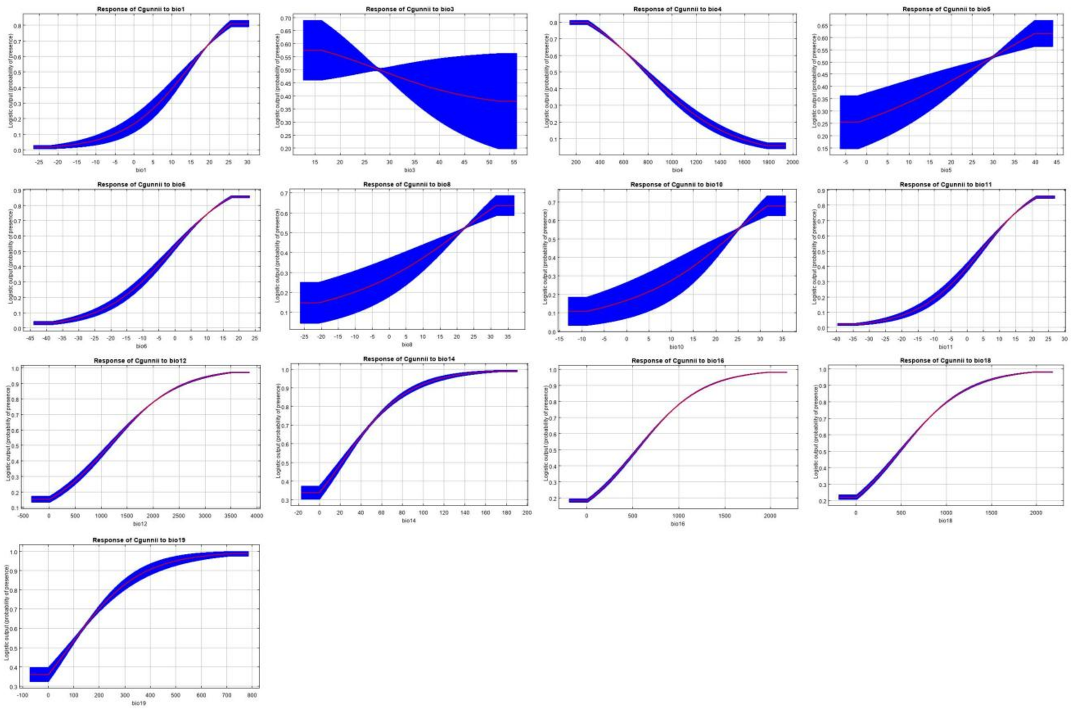

Supplementary figure S5-4. Response curves of 13 environmental variables to *C. gunnii* under current climate conditions.

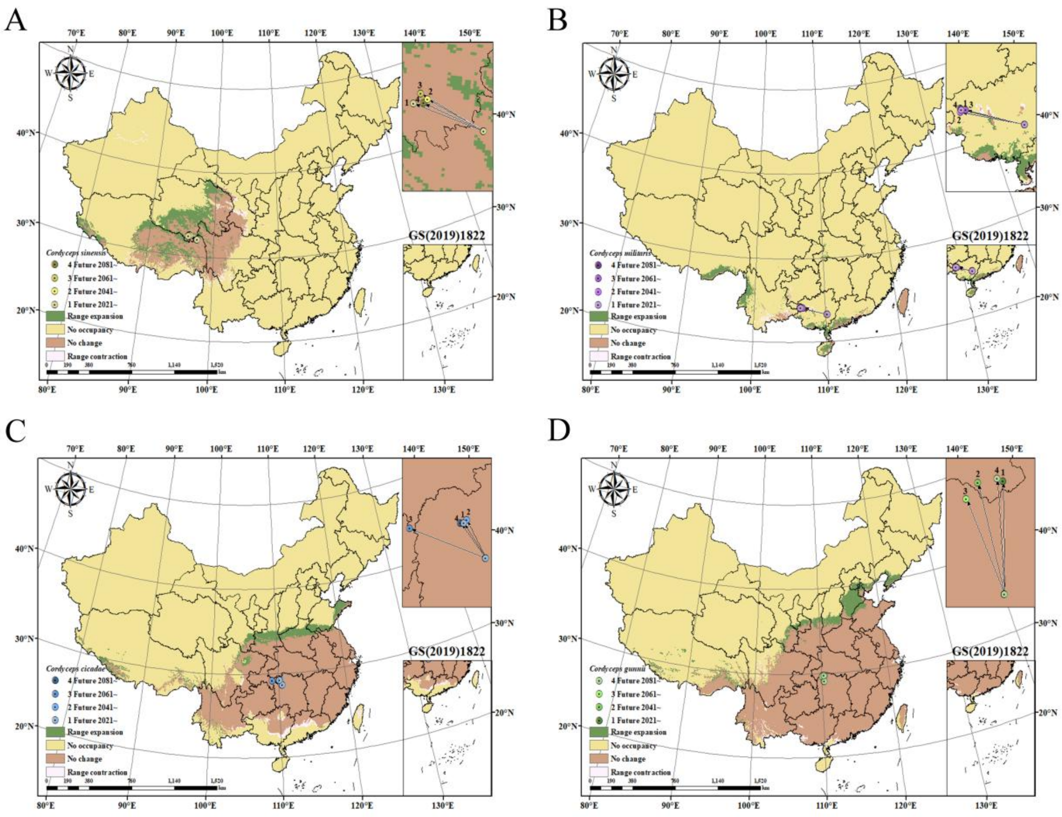

Supplementary figure S6. Four future periods (2021-2040, 2041-2060, 2061-2080, 2081-2100) direction of centroid migration in suitable areas of *C. sinensis, C. cicadae, C. militaris and C. gunnii.*

Supplementary table

Supplementary table 1

Supplementary table 1S. Optimization results of MaxEnt software parameters.

Species	FC	RM	Mean_AUC_ratio	Omission_rate_at_5%	delta_AICc
C.sinensis	LQ	0.1	1.93	0.04	0
C.cicadae	QP	0.7	1.95	0	0
C.militaris	LQ	0.2	1.70	1	0
C.gunnii	P	2.3	1.89	0.14	0
